# Characteristic System Time Scales Can Influence the Collective Sequence Development of Nematically Ordered Copolymers

**DOI:** 10.1021/acs.macromol.4c01047

**Published:** 2024-10-15

**Authors:** Ryan L. Hamblin, Zhongmin Zhang, Kateri H. DuBay

**Affiliations:** † Department of Chemistry, University of Virginia, Charlottesville, Virginia 22903, United States; ‡ Department of Chemistry, University of North Carolina at Chapel Hill, Chapel Hill, North Carolina 27599, United States

## Abstract

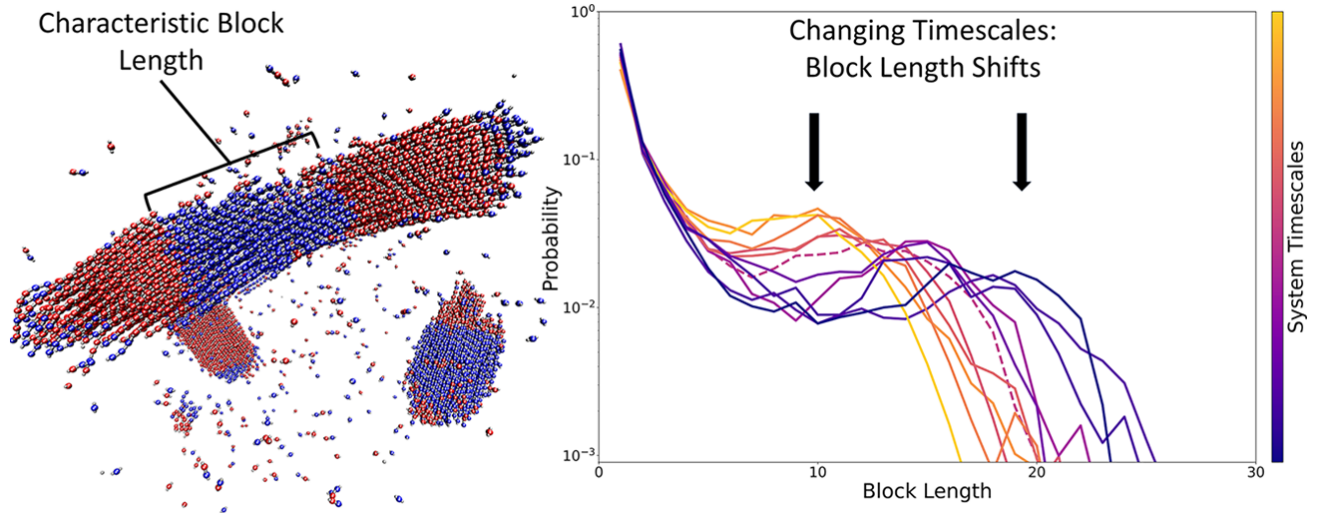

The sequence of copolymers is of significant importance to their material properties, yet controlling the copolymer sequence remains a challenge. Previously, we have shown that polymer chains with sufficient stiffness and intermolecular attractions can undergo an emergent, polymerization-driven nematic alignment of nascent oligomers during a step-growth polymerization process. Both the extent of alignment and the point in the reaction at which it occurs impact the kinetics and the sequence development of the growing polymer. Of particular interest is the emergence of a characteristic block length in the ensemble of sequences, resulting in unusually peaked block length distributions. Here we explore the emergence of this characteristic block length over time and investigate how changes in activation energy, solution viscosity, and monomer density influence the sequence and block length distributions of stiff copolymers undergoing step-growth polymerization. We find that emergent aggregation and nematic ordering restrict the availability of longer chains to form bonds, thereby altering the propensity of chains to react in a length dependent fashion, which changes as the reaction progresses, and promoting the formation of chains and blocks of a characteristic length. Further, we demonstrate that the characteristic length scale which emerges is sensitive to the relative time scales of reaction kinetics and reactant diffusion, shifting in response to changes in the activation energy of the reaction and the viscosity of the solvent. Our observations suggest the potential for biasing characteristic lengths of sequence repeats in stiff and semiflexible copolymer systems by targeting specific nonbonded interactions and reaction kinetics through the informed adjustment of reaction conditions and the selection or chemical modification of monomer species.

## 1 Introduction

Developing general synthetic methodologies for sequence biasing remains an open challenge in polymer science, as current methods for sequence control in synthetic copolymers lack the high degree of specificity or, crucially, the ease of application found in their biological counterparts.^1−6^ Sequence control in synthetic polymers opens up a host of potential applications, both as macromolecular carriers of information, providing a route for developments in nonbiological molecular information storage and synthetic biology,^4,7,8^ and as highly functional materials due to the direct dependence of material properties and macromolecular morphologies on the polymer sequence. Various studies have shown that mechanical and rheological properties,^9,10^ thermal and ionic conductivity,^11−14^ glass transition and crystallinity,^15−17^ morphology and phase behavior,^10,13,18,19^ coacervation and electrostatic interactions,^20,21^ optoelectronic properties,^22,23^ and even sound and vibrational damping^24,25^ are influenced by the primary monomer sequence, as evidenced by comparisons between polymers with different sequences but the same overall fractional monomer content.^9,13,14,17,19,20^

Our previous work has shown that differences in nonbonded attractive interactions that approach the strength of thermal fluctuations (∼*k*_B_*T*), such as those arising from differences in monomer–solvent affinities, can significantly influence the sequences obtained from a solution-based step-growth polymerization.^26−28^ This sequence influence is due to the growth of nascent oligomers, which alters the free energy of mixing, as explained by Flory–Huggins solution theory, and can lead to a microphase separation among the reactants as the reaction proceeds.^29^ This local demixing occurs as oligomers lengthen with attractive interactions driving the formation of emergent concentration heterogeneities in the local reaction environment, altering the likelihood of sequence additions. The chain stiffness of the growing oligomers, as characterized by their persistence lengths, also influences both the sequence development and the self-assembly of the resulting aggregates by altering the behavior of the emergent demixed microphases.^27,28,30^ Oligomers with a longer persistence length can form highly ordered, nematically aligned phases as the reaction proceeds, giving rise to what we term a self-templating effect that alters subsequent chain growth. Both the length of the chains formed and their block lengths, i.e., the lengths of sequences of repeated monomers of a single type, are influenced by this effect. Such templating behaviors lead to the development of characteristic lengths, in which the reaction is biased toward the formation of particular chain and block lengths.

In this work, we investigate the emergence of these characteristic lengths in time, examining how the bonding behaviors of stiff-chain copolymers differ from those with more flexible chains, leading to altered sequence ensembles. Further, we explore how features of the system which influence reaction kinetics and the relevant characteristic time scales within the system alter these bonding behaviors at different stages of the reaction, thereby impacting the chain and block length statistics. To this end, we vary the activation energy of the reaction, the solution viscosity, and the initial monomer density to examine their effects on the kinetics of the reaction, the alignment of the growing oligomers, and the resulting chain and sequence statistics. We find that the characteristic length scale we observe shifts in a predictable fashion in response to the characteristic system time scales defined by diffusion and reaction kinetics in the system.

We begin by describing the model and simulation methods in Section 2. In Section 3.1 we examine how the sequence self-templating phenomenon we observe responds to changes in the reaction conditions, including the nonbonded interaction strength, reactant concentration, monomer reactivity, and solvent viscosity. In Section 3.2 we relate these reaction conditions to characteristic time scales of the system, noting the variation in the onset of sequence templating and the resulting characteristic length scale shifts predictably in response to changes to the rates of reaction and monomer diffusion. In Section 3.3 we describe how this self-templating sequence behavior emerges in time, arising as a consequence of the simultaneous impact of emergent demixing and nematic ordering with an associated change in bonding behaviors in the system. In Section 3.4 we explore the nucleation and growth of oligomer aggregates, examining how aggregate morphology drives these changes to bonding behavior within the aggregated phase in a manner that depends on the length and stiffness of the reacting chains. We then briefly conclude in Section 4, discussing our observations in connection to analogous phenomena and in the context of conjugated, stiff-chain copolymers and their aggregates.

## 2 Methods

### 2.1 Monomer Structure and Interactions

To simulate an irreversible, step-growth polymerization, we make use of a model previously developed in ref (26) and expanded in subsequent works.^27,28,30^ The model utilizes a coarse-grained representation, with bifunctional monomers represented as simplified spherical “beads”. Each bead is composed of three particles: a central particle connected to two external particles via harmonic bonds. These harmonic bonds control the spatial and angular separation of the three particles in the bead, modeling the internal configuration of the monomer and determining the chain stiffness. The central particle of each monomer bead contains the majority of the mass and determines the monomer species, denoted either **A** or **B** to represent the two comonomers taking part in the copolymerization. The external particles represent the reactive functional groups and form bonds with the external particles of the other monomers when the reaction conditions are met.

Unbound monomers interact with one another via two potentials, the first of which is a modified Lennard-Jones (LJ) potential acting on the central particles of the monomer. The repulsive forces in the LJ potential are kept constant between all monomer types, maintaining fixed monomer sterics. The attractive portion of the LJ potential is the source of all nonbonded attractions in the system and has a strength determined by the parameter ε_*ij*_. These attractive interactions are then defined to take different values based on the species *i* and *j* pairs of the interacting monomer pair, denoted as ε_AA_, ε_BB_, and ε_AB_. This work specifically explores the case of symmetric like-monomer attractive interactions, corresponding to the selection of ε_AA_ = ε_BB_ ≡ ε_AA,BB_. As we are interested in the impact of relatively small differences in attractive interaction strengths, we chose ε_AA,BB_ = *k*_B_*T* and ε_AB_ = 0. The external particles of each monomer bead also interact with each other via a very short-range, isotropic, and purely repulsive potential, designed as an adjustable addition to the activation energy of the reaction. Explicit analytical forms of the nonbonding interaction potentials are provided in eqs S1 and S2 in the SI.

### 2.2 Simulation Progression and Reaction Mechanism

**Figure 1:**
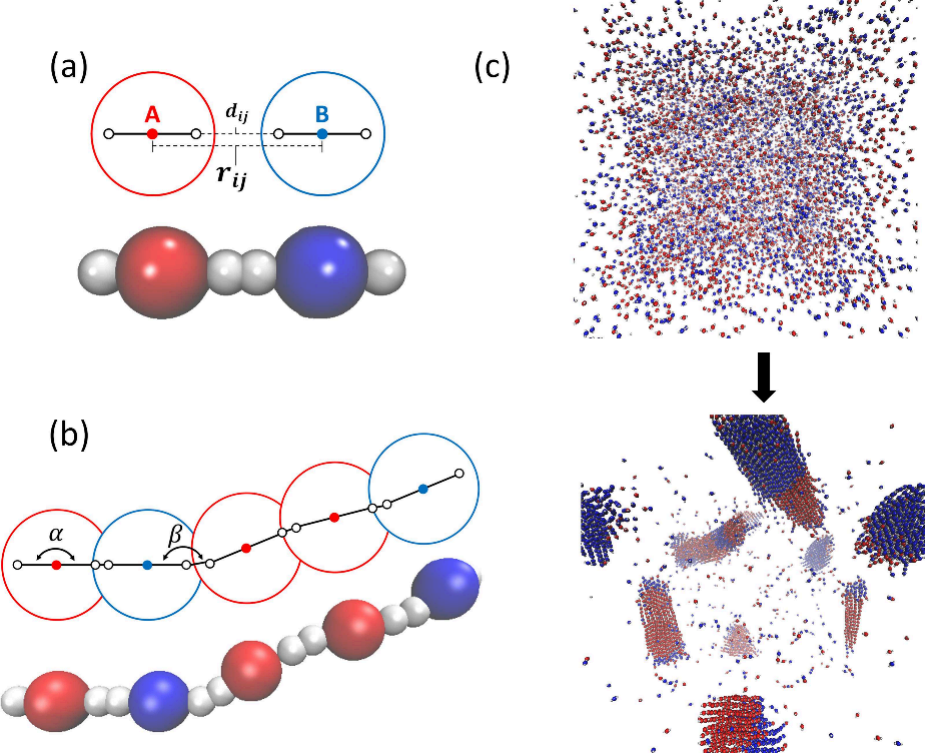
Coarse-grained model schematic and reaction visualization. (a) Schematic and associated visualization of each monomer type in the copolymerization simulation. Monomers are represented as coarse-grained spherical beads containing three particles as shown. Central particles define the monomer type, either A or B, while the external particles represent reactive moieties. Both the center-to-center distance *r*_*ij*_, which controls the intermolecular interactions via the LJ potential, and the interaction particle distance *d*_*ij*_, which determines the activation energy and bonding events, are shown. When *d*_*ij*_ ≤ 0.2σ, an irreversible bond is formed. (b) A growing oligomer chain. Within the schematic, the intramonomer angle α and the intermonomer angle β are shown, each with associated angular harmonic potentials. The strength of the α angular potential determines the stiffness of the chain. (c) Visualization of the initial and final system states for a single step-growth copolymerization simulation.

Reaction events are modeled by defining a specific cutoff distance *d*_bond_. When the external particles of two monomers cross within this distance, overcoming both the fixed steric repulsions from the central particles and the additional repulsions between the external reactive particles, an irreversible harmonic bond is formed (Figure 1a). Each functional group is capable of forming one such new bond, and the reaction thus proceeds through a step-growth mechanism.

Each simulation begins with all monomers unbound and randomly distributed throughout the simulation domain. The reaction then progresses via Langevin dynamics^31^ until reaching a monomer conversion ratio of *p* = 0.9, indicating 90% of monomers have reacted. Sampling the system via the Langevin equation not only produces realistic dynamics from the potentials in the system but also incorporates a viscous drag force and random collision forces characteristic of the solution state and serves as a thermostat for the system. Adjusting the parameters of the Langevin equation allows us to reproduce solution-state dynamics for a range of different solution viscosities (see Sec. S1.3 in the SI for details). It is important to note that, for all simulation parameters explored in this work, monomers remain well-dispersed in the solution phase prior to reaction, only beginning to aggregate as the reaction proceeds and oligomers lengthen due to the consequences of Flory–Huggins theory.^29^

### 2.3 Quantifying Chain Stiffness and Nematic Alignment

As a quantitative measure of the chain stiffness, we consider the persistence length *l*_*p*_ defined as
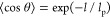
1where *l* is the contour length distance between two points on the polymer chain and θ is the angle between the tangent lines drawn at each of these two points. An average is taken over an equilibrium ensemble of configurations at each contour length distance of *l*. All simulations in this work were run with an angular potential chosen to produce persistence lengths of *l*_*p*_ = 16.5 monomer units (see Sec. S1.5 in the SI for details). This corresponds to the highest values of *l*_*p*_ explored in our previous work.^30^

In order to characterize the local alignment of oligomer chains, we define a local nematic ordering parameter  as shown:
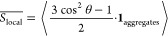
2Here θ is the angle between the orientation of a single monomer within the aggregate and its local director, a vector taken as the average of all monomer orientations within the local environment, i.e., within 2.5σ of the monomer. The ensemble average, over each monomer in the system, is taken of the second order Legendre polynomial *P*_2_(cos(θ)) multiplied by an indicator function **1**_aggregates_. The function **1**_aggregates_ takes the value 1 for monomers in aggregates and 0 elsewhere. For this calculation, a monomer is considered to be within an aggregate if there are 12 or more neighboring monomers within a distance of 2.5σ. So defined,  is closely related to the standard liquid crystal ordering parameter,^32^ differing only in the addition of the indicator function which excludes nonaggregated chains and in the definition of the local director as the average orientation within the local environment. Just as in the standard nematic order parameter,  for perfectly isotropic chain orientations and approaches  for aggregates of fully nematically aligned chains.

## 3 Results and Discussion

### 3.1 Reaction Conditions Shift the Characteristic Block Length

In our previous work, we showed how copolymer chains of sufficient stiffness and nonbonded attractions yield an emergent characteristic block length, which alters the distribution of block and chain lengths and reduces their dispersity.^30^ Here we begin by considering the extent to which this sequence biasing phenomenon may be influenced by other properties of the reaction, independent of the chain stiffness.

**Figure 2:**
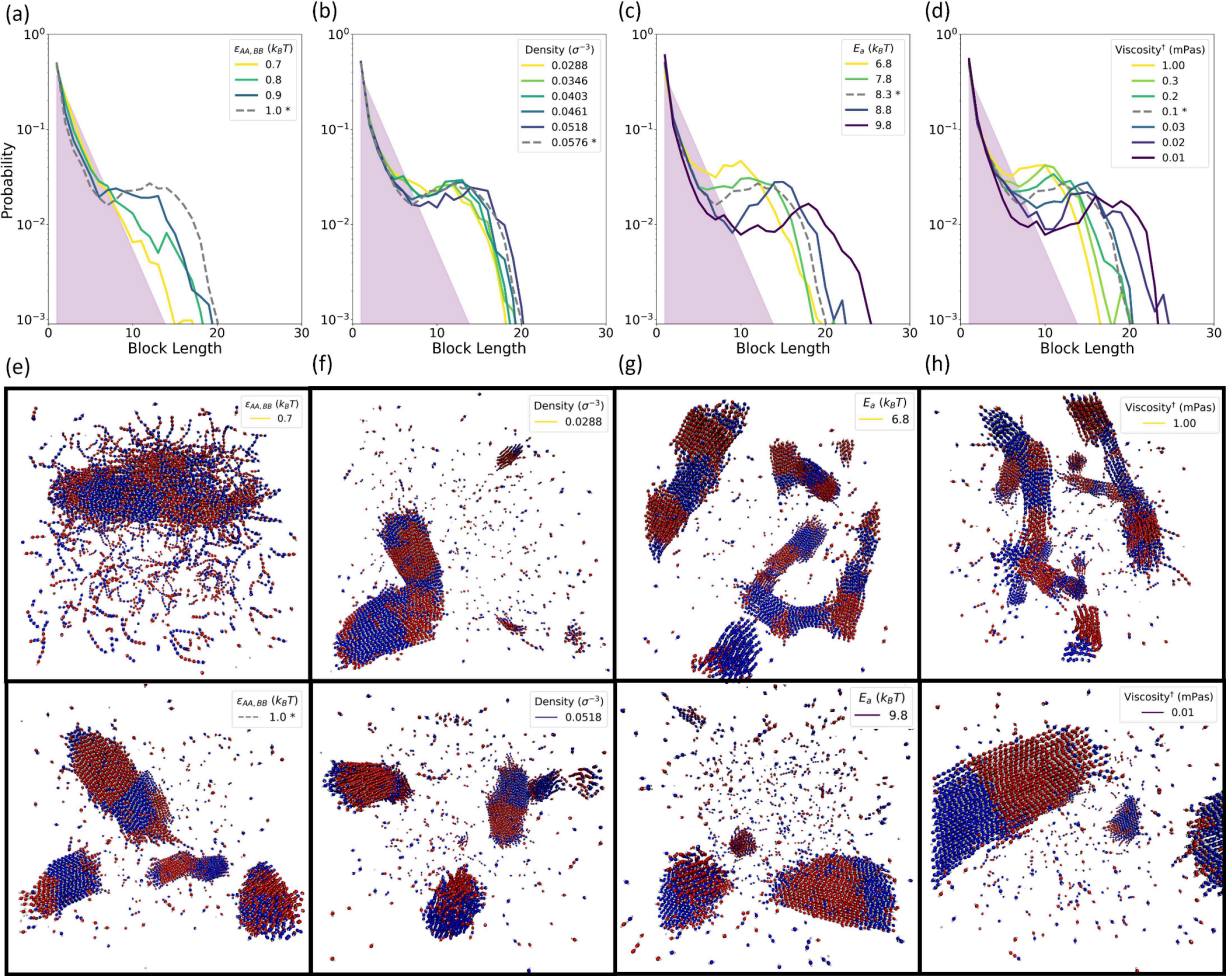
Block length distributions vary with attraction strength, monomer density, activation energy, and viscosity. (a–d) Block length distributions of all-**A** or all-**B** blocks are shown for the copolymerization of stiff chains (*l*_p_ = 16.5) at a reaction extent of *p* = 0.9. Results are shown here for variations in (a) like-monomer attraction strength, (b) initial monomer density, (c) activation energy, and (d) solvent viscosity. The gray, dashed lines in each plot are identical and display the block length distribution found under the standard conditions described in ref (30). These conditions are indicated with * in the legends, which also specify the values of the conditions held constant in (a–d). Each distribution is obtained from three independent simulation trials at a reaction extent of *p* = 0.9. The shaded purple region corresponds to the results expected from Markov statistics (see Sec. S3 in the SI) for the case with ε_AA,BB_ = 0.7*k*_B_*T* pictured in (e), where we observe the onset of polymerization driven assembly behaviors. (e–h) Visualization of representative structures under select conditions from (a–d). Each column corresponds to changes in the same property. The center row showcases conditions with the minimal block length distribution shift, while the bottom row shows conditions with a maximum shift in the block length distribution. Specific parameters for the visualizations are indicated by the inset legend, which correspond to those in the block length distributions in (a–d).

To investigate the dependence of the observed emergent characteristic all-A or all-B block length on factors other than the persistence length, we varied the strength of nonbonded attractions, the initial bulk density of the monomers, the activation energy of bond formation, and the viscosity of the implicit solvent. We therefore ran a series of simulations which modified one of each of these variables over a range of values, while keeping the other variables fixed. For additional details on how these variables were adjusted in the simulation, refer to Sec. S1.3 and S1.4 in the SI. Figure 2 shows the results of these simulations, examining the response of the block length distribution to changes in each of these parameters in Figure 2a–d and showcasing sample structures for two conditions in each case in Figure 2e–h. The block length distributions in Figure 2a–d are contrasted against a distribution obtained from Markov statistics, as described in section S3 in the SI. In the plots shown in Figure 2a–d, the results for our set of standard conditions in previous studies^26,30^ are the same across plots and indicated with a gray dashed line. Additional results showing the relative proportion of oligomer bonding, namely, bonding involving oligomers of length ≥3 monomers, alongside block length distributions under the conditions shown in Figure 2, are provided in Figure S9 in the SI.

The dependence of the emergent block length on each of these variables differs and broadly falls into three categories. The first of these is the behavior of the system in response to a reduction in the strength of nonbonded attractions, namely, to a reduced value of ε_AA,BB_, which governs the strength of intermonomer attractions. Here we see that the characteristic block length (Figure 2a) rapidly disappears from the system with a reduction of the like-monomer attractions of only 0.3*k*_B_*T*. This result highlights the absolute dependence of the observed phenomenon on the emergent aggregation and assembly driven by effective nonbonded like-monomer attractions. The reduction in attraction strength modifies the phase behavior so that the cohesive energies are insufficient to overcome the entropic cost of aggregation until very late stages of the reaction, when chains of sufficient length have formed (Figure 2e, top). With the impact of the emergent aggregation and phase separation thus greatly reduced, the resulting block length distribution collapses toward the purple-shaded distribution expected from Markovian statistics, which describes the sequence behavior when the probability of each additional monomer in the chain depends only on the identity of the preceding monomer (Figure 2a). By contrast, increased attractions result in aggregation and assembly occurring much earlier in the reaction, meaning a greater proportion of bond formation and sequence development occurs after oligomers begin to assemble. In such instances, the impact of collective behaviors on sequence is more robust, and the associated block distribution shift away from the Markovian distribution is more pronounced.

In contrast to the dramatic onset of new phase and sequence behaviors seen in response to small variations in attractive energies, the response to variation in the initial monomer density is minute. Even at a 50% reduction in the initial monomer density, the distribution of block lengths (Figure 2b) remains largely unchanged, with the peaked distribution indicative of a characteristic block length remaining, as can be seen in the snapshots in Figure 2f. It should be noted that polymerization induced phase separation as a whole depends upon reactant density, and we therefore necessarily restrict our exploration of density values to be low enough that reactants are initially well solvated but large enough to undergo sufficient aggregation at oligomer lengths reached in the simulation as the reaction proceeds. Within this density regime, however, the aggregation behavior and emergence of the observed characteristic block length are not sensitive to the absolute rate of the reaction, a reactant density dependent property.

The third type of dependence we observe in Figure 2 is in response to variations in the relative, rather than absolute, kinetic rates within the system, through changes to the activation energy of the reaction (Figure 2c) and the viscosity of the implicit solvent (Figure 2d). While a characteristic block length remains in evidence across variations in these time scales, changes to these variables shift its length. Changing these conditions also alters the timing and extent of oligomer bonding, i.e., bonding that involves at least one chain of length ≥3 monomers in the system (see Figure S9 in the SI for oligomer bonding details). Not only do the block length distributions show similar shifts under variation to each of these quantities, but changes to the structure and morphology of the aggregates appear analogous as well. Conditions leading to a shorter characteristic length show smaller and more numerous aggregates (Figure 2g and h, top), while single large aggregates dominate the systems in which conditions lead to a longer block length (Figure 2g and h, bottom).

### 3.2 Block Length Distribution Shifts Correspond to Changes in Relative Kinetic Time Scales

To better quantify the observed changes in characteristic block length biasing, we now consider the effective reaction and diffusion time scales in the system in terms of the activation energy of the reaction and the solvent viscosity.

In order to define a characteristic time scale for the reaction, τ_R_, we consider the inverse of the effective rate constant for polymerization in the absence of aggregation behaviors, *k*_eff_, taking τ_R_ ≡ *k*_eff_^–1^.^33^ The kinetics for our model system have been worked out in detail previously,^28^ allowing us to connect the measured activation energy, *E*_a_, to our effective rate constant and thus to our reactive time scale, τ_R_.

To characterize the impact of solvent viscosity on the diffusional time scale of the reactants, we make use of the Stokes–Einstein relation to determine a diffusion coefficient, *D*, in terms of the solvent viscosity, η, and the size of an individual monomer, σ. Specifically, we define the time scale of diffusional motion using τ_D_ ≡ σ^2^/*D*, i.e., the time needed for a monomer to diffuse its own length.

It should be noted that we expect the behaviors we observe to depend on the relative balance of these two time scales, τ_D_/τ_R_, and not on the values of the time scales themselves. As such, the trends we observe should not be influenced by constant factors that alternative definitions of τ_D_ and τ_R_ might introduce, so long as the functional forms of their dependence on the tunable parameters in our model, τ_D_(η) and τ_R_(*E*_a_), remain consistent.

**Figure 3:**
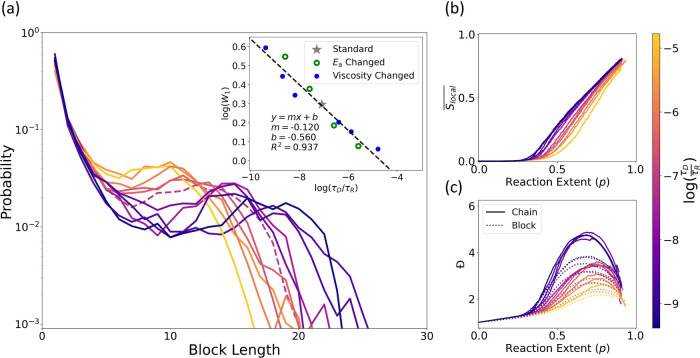
Block length distribution shift and characteristic time scales. (a) Block length distributions of all-**A** or all-**B** blocks observed at a reaction extent of *p* = 0.9, colored according to the value of log(τ_D_/τ_R_), the logarithm of the ratio of the characteristic diffusive and reactive time scales for the simulation. Inset: A log–log plot of the Wasserstein distance, *W*_1_, vs the ratio τ_D_/τ_R_. The Wasserstein distance was calculated between the observed distribution of block lengths and the distribution expected from Markov statistics (see the SI for details). The result from standard simulation conditions, i.e., those used in ref (30) and the gray dashed lines in Figure 2, is shown in gray with a star marker. Simulations in which viscosity was varied are shown in blue, and simulations in which activation energy was varied are shown in green. A linear regression was performed, and the resulting regression equation and fit-line are shown. (b) Local nematic ordering parameter, , as a function of reaction extent. (c) Chain length (solid line) and block length (dotted line) dispersity, *Đ*, as a function of reaction extent. All data was obtained from three independent simulation trials for each parameter set.

With the chosen definitions of τ_D_ and τ_R_ in hand, we are able to map our variations in activation energy and solvent viscosity to the relative balance of diffusional and reactive time scales in our system. Figure 3 shows the combined results of simulations from Figure 2c and d, in which either solvent viscosity or reaction activation energy was varied, as quantified by the ratio of time scales, τ_D_/τ_R_, indicated by line coloration. The extent of the block distribution shift in Figure 3a clearly corresponds to a shift in the ratio of these characteristic time scales, τ_D_/τ_R_, as can be seen in the color progression from left to right.

To quantify the observed shift in the block length distribution, we make use of the first-order Wasserstein distance,^34,35^*W*_1_, as a measure of the statistical distance between our observed block length distribution and the block length distribution expected if sequence development obeyed Markovian statistics.^26,27^ For two discrete, univariate probability distributions *p*(*x*) and *q*(*x*) with cumulative distribution functions *P*(*x*) and *Q*(*x*), the first-order Wasserstein distance may be defined^34,35^ as
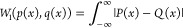
3

Utilizing this statistical distance, *W*_1_, we find a direct quantitative relationship (Figure 3a, inset) between the degree of distribution shift and the ratio of characteristic time scales in the system. Alongside the clear correspondence between block length distributions and coloration in the main panel of Figure 3a, this quantitative relationship suggests that the effect of changes to both the activation energy (Figure 2c) and solvent viscosity (Figure 2d) can be captured in a unified way through τ_D_/τ_R_.

The onset of nematic ordering, characterized by  in Figure 3b, is similarly impacted by changes to the balance of characteristic time scales, as are the chain and block length dispersities shown in Figure 3c. Each of these features develops earlier or later in the reaction and, to a greater or lesser extent, at lower or higher τ_D_/τ_R_, respectively. This shared response to the value of τ_D_/τ_R_ highlights the connection between the onset of nematic ordering, the non-monotonic progression of dispersity, and the block length distribution and indicates the importance of the balance of the reaction and diffusion time scales to each of these features.

The trends observed with respect to the reaction barrier and the diffusion rate can be readily explained if we consider that a small nucleus of nascent chains grows into a crystallite by reacting with other monomers and oligomers that match the growing nuclei, in terms of both its monomer identities and its overall length. If the reaction is slowed down, relative to the relaxation time of oligomers tumbling about in the system, then the system has more time to settle into energetically favorable prereaction aggregates before the formation of additional polymer bonds, leading to an impressive alignment in terms of the overall oligomer length and the block lengths within each crystallite. This relative slowing of the reaction time, as compared to the system’s relaxation time, can be achieved by increasing the reaction barrier or by increasing the diffusion rate, and the block length distributions in Figure 2c and d and Figure 3a clearly show these trends. As the reaction rate slows with respect to the rate of reactant diffusion, the system has additional time to relax toward lower energy configurations as chains extend. As a result, phase separation, aggregation, and nematic alignment happen earlier with respect to the reaction extent (Figure 3b). This earlier aggregation and ordering leads to an earlier emergence of bonding involving longer than average chains and a greater reduction in long chains bonding with other long chains late in the reaction (see Figure S8 in the SI for details). Such variations in bonding behaviors at different reaction extents lead to the non-monotonic dispersity behavior (Figure 3c).

Together, the results in Figures 2 and 3 demonstrate a sequence biasing behavior which arises from phase changes driven by the reaction itself, producing a characteristic block length which may be directly influenced through changes to the diffusivity and reactivity of the reacting species. We note here that it is not immediately clear why the relationship between *W*_1_ and τ_D_/τ_R_ takes the functional form that we observe here, and the underlying theoretical basis behind this relationship warrants further investigation in future work. Nevertheless, these results clearly demonstrate that the shift in block length distribution, captured by the descriptive statistic *W*_1_, is correlated with changes to the balance of the relevant system time scales described by τ_D_/τ_R_. In our subsequent analyses, we will examine the origins of the characteristic block length, showcasing the relationship between chain length dependent bonding behaviors and the development of nematic and sequence ordering in the system.

### 3.3 Characteristic Block Length Arises from Emergent Changes in Bonding Behaviors

**Figure 4:**
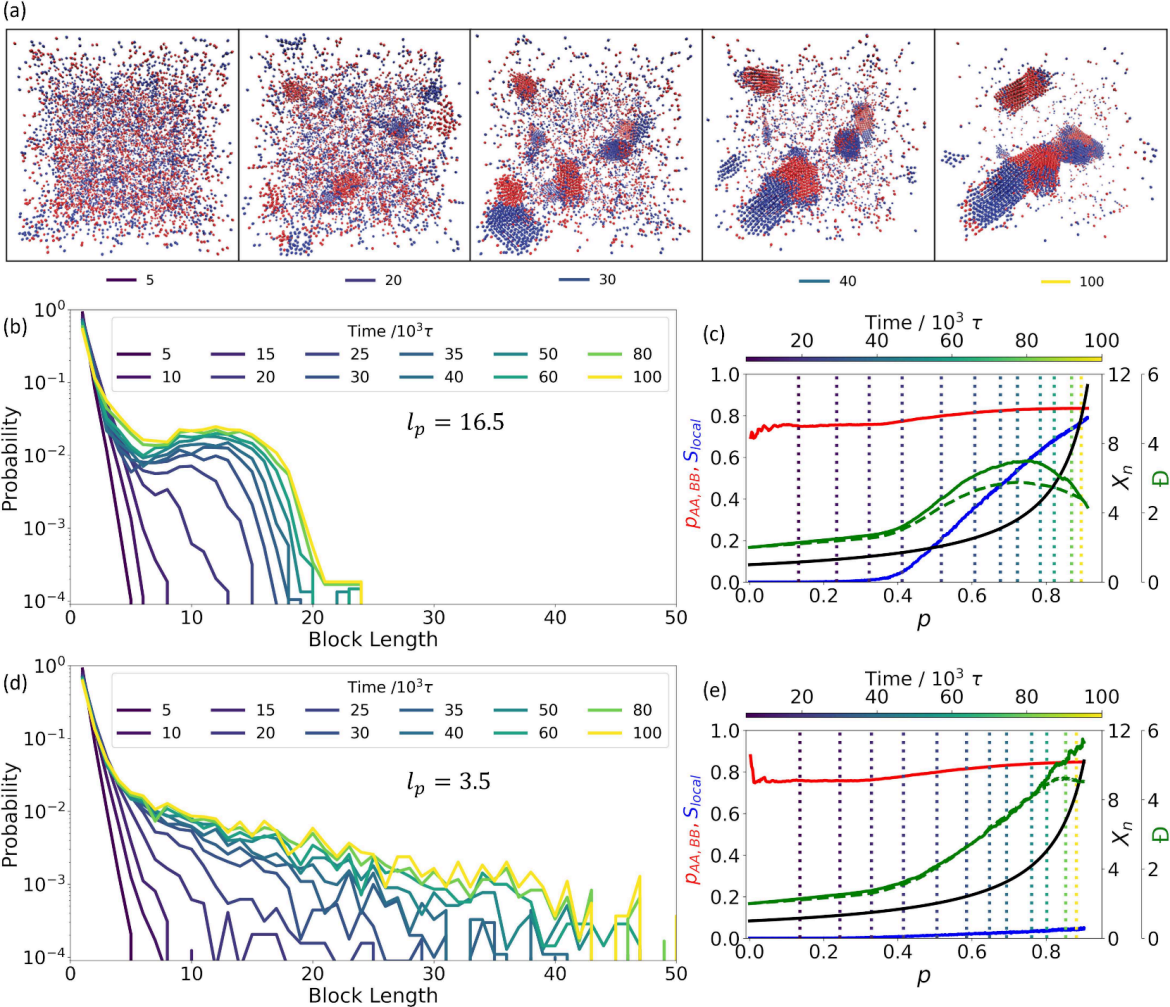
System structures and block length distributions during copolymerization of semiflexible polymers. Results from reactive simulation of two different persistence lengths, *l*_p_, for ε_AA,BB_ = *k*_B_*T* and ε_AB_ = 0. In (a–c) *l*_p_ = 16.5 monomers, and in (d, e) *l*_p_ = 3.5 monomers. (a) Characteristic snapshots of structures forming within a single simulation are shown at five different points in the reaction. (b, d) The block length distributions for (b) stiff chains and (d) flexible chains are plotted at 12 different time points in the reaction. (c, e) The change of sequence neighbor probability *p*_AA,BB_ (red line), order parameter  (blue line), degree of polymerization *X*_*n*_ (black line), and chain (solid green line) and block length (dashed green line) dispersity *Đ* are shown as functions of reaction extent, *p*, for (c) stiff chains and (e) flexible chains. The chosen times presented in (a), (b), and (d) are indicated with vertical dotted lines, with coloration corresponding to the simulation time.

We now investigate the development of this sequence biasing in time, exploring how the characteristic block length arises as a consequence of a nematic ordering transition that changes the bonding behavior of oligomers within aggregates that are nucleated early in the reaction. We begin by examining, in Figure 4, the time evolution of both structural snapshots and block length distributions for the reaction of stiff and flexible A,B-copolymers under our standard simulation conditions (shown in gray dashed lines in Figure 2a–d and the dashed line in Figure 3a).

#### Early Stage (*p* ≤ 0.4)

The simulation snapshots in Figure 4a clearly demonstrate the gradual onset of aggregation coupled with a microphase separation into **A**-rich and **B**-rich domains. The concurrent impact of these emergent aggregation processes on sequence can be seen in the time progression of the block length distribution, as shown in Figure 4b. At early times, the distribution of block lengths closely matches the geometric distribution expected for a polymerization process governed by Markovian statistics. However, by the time aggregates appear in Figure 4a at 20 × 10^3^τ (*p* ≈ 0.4), a longer tail is observed in the distribution in Figure 4b, indicating the presence of longer-range ordering in the oligomer sequences—ordering that persists past the nearest sequence neighbors.

Other properties of the reaction kinetics, shown in Figure 4c, are also impacted by the emergent aggregation that happens around 20 × 10^3^τ (*p* ≈ 0.4): both chain-length dispersity (solid green line) and degree of polymerization (black line) increase in a nonlinear manner—a sign of the breakdown in standard kinetic behaviors driven by this emergent reactant heterogeneity.^26,28^ Longer-than-average oligomers are more prone to aggregate and therefore to encounter and react with other long oligomers, which increases the dispersity and speeds chain growth. Aggregation also noticeably alters the observed probability, *p*_AA,BB_, of a repeat sequence pair in the ensemble of oligomer sequences (either **AA** or **BB**). The value of *p*_AA,BB_ (red line) remains relatively consistent prior to aggregation, increases upon aggregation, and remains consistent at the increased value for the rest of the reaction. The impact on the sequence pair probability is a consequence of the emergence of the **A,B** microphase separation, which enriches the concentration of the like-monomer species within the local environment and leads to an increased likelihood of encounter and reaction between monomers of the same type.^26^

#### Middle Stage (0.4 < *p* ≤ 0.7)

Up to this point in the reaction, both stiff chains (Figure 4a–c) and flexible chains (Figure 4d,e) behave in largely the same fashion with one notable exception. For stiff chains, orientational alignment of oligomers, characterized by the order parameter , begins to increase with the onset of aggregation and increases continuously throughout the remainder of the reaction (Figure 4c), indicating a nematic transition that is absent among the flexible chains (Figure 4e). Early stiff chain aggregates are predominantly isotropic at 20 × 10^3^τ (Figure 4a), as can be seen in the low value of  at that time (Figure 4c, *p* ≈ 0.4). By 30 × 10^3^τ (*p* ≈ 0.6); however, the chains within each aggregate show signs of significant ordering (Figure 4a), with  continuing to increase as chains align and the crystallites grow.

Concurrent with this nematic ordering, between 20 × 10^3^τ and 30 × 10^3^τ, a peak forms in the block length distribution of the stiff chains (Figure 4b), indicating the emergence of a characteristic block length, which remains absent from the equivalent distribution of flexible chains (Figure 4d). This distributional peak shifts to the right and becomes more distinct as the reaction proceeds, and by 40 × 10^3^τ (*p* ≈ 0.7) the characteristic block length observed at the end of the reaction is well established.

#### Late Stage (0.7 < *p* ≤ 0.9)

Additional bonding past this stage generally forms more sequences of that characteristic length rather than increasing it so that the distribution curves begin to coalesce. Importantly, the decrease in dispersity, shown in Figure 4c, also occurs at this point, arising as a consequence of the nematic alignment once sufficient ordering is present in the system (). This decrease indicates that chains that are longer than the average are preferentially reacting with chains that are shorter than the average, reducing the overall dispersity. This two-stage, length dependent bonding behavior is unique to the aggregates formed from stiff chains. Flexible chains demonstrate no such decrease in dispersity, and the preference for long oligomers to bond other long oligomers, which spurred the initial nonlinear increase in dispersity, continues throughout the reaction (Figure 4e).

#### Chain Length Dependent Bonding

**Figure 5:**
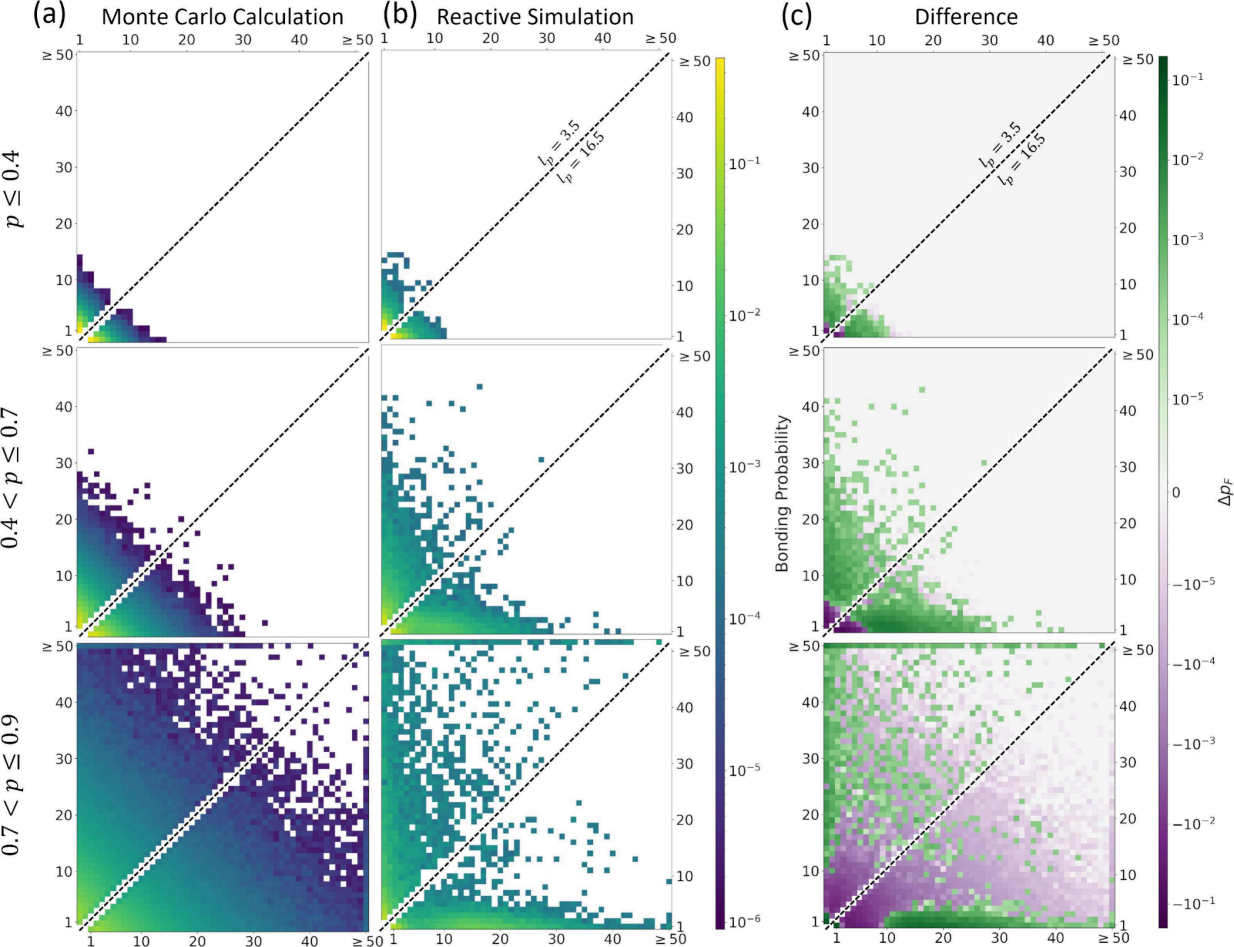
Chain-length dependence of bond formation. These plots show the probability that chains of different lengths will form a bond during different periods of the reaction. Each row corresponds to bond events sampled during a different range of the simulated reaction extent: *p* ≤ 0.4; 0.4 < *p* ≤ 0.7; and 0.7 < *p* ≤ 0.9. Within each plot in (b) and (c), the upper-left half shows the values for flexible chain systems (*l*_*p*_ = 3.5) and the lower-right half shows the values for stiff chains (*l*_*p*_ = 16.5). Probabilities in column (a), *p*_F_, were determined from 250 simple Monte Carlo calculations of the ideal Flory behavior, in which no spatial or stiffness information is taken into account, and thus all chains have an equal probability of reacting at each step in the reaction, and the two halves are therefore mirror images (see the SI for details). Probabilities in column (b), *p*_observed_, were determined from actual bonding events observed in our reactive Langevin dynamics simulations from three independent trials for both flexible and stiff chains. In column (c), the difference, *Δp*_F_, is shown between the observed probability in (b) and the probability which occurs under ideal Flory behavior in (a). All probabilities are normalized by the number of bonding events within the stated reaction extent range.

To further quantify the length dependent bonding behavior we observe, we present in Figure 5 histograms of the likelihood of bonding between chains of different lengths over the early (*p* ≤ 0.4, top row), middle (0.4 < *p* ≤ 0.7, middle row), and late (0.7 < *p* ≤ 0.9, bottom row) stages of the reaction. Figure 5a shows the results from trials of a simple, idealized Monte Carlo calculation designed to reproduce the sequence statistics expected under conditions where Flory’s equal reactivity principle^29^ holds, namely, where all extant chains are equally likely to form a bond (see Sec. S2 in the SI for details). While such a calculation fundamentally cannot account for spatial heterogeneities of reactants or the stiffness and orientations of the nascent chain, it is nevertheless an effective implementation of the assumptions of the equal reactivity principle, which similarly does not capture these details. Figure 5b shows the results for our model system under both flexible (upper-left diagonal) and stiff (lower-right diagonal) cases. Figure 5c shows the difference, *Δp*_F_ ≡ *p*_observed_ – *p*_F_, in the bonding probability between our observations, *p*_observed_, and the ideal Flory behavior, *p*_F_.

Under equal reactivity (Figure 5a), early stage bonding is dominated by monomer–monomer reactions, and only late in the reaction do longer chains begin to emerge. Even late in the reaction, the majority of bonding involves short chains of length ≤3, and only very rarely do two longer oligomers react. It should be noted here that there is no difference in bonding probabilities arising from differences in chain stiffness in Figure 5a, as by definition equal reactivity, which is taken as the basis of these Monte Carlo calculations, does not permit such properties of the larger chain to affect the bonding behavior. The chain persistence length therefore does not enter into this calculation, and the equivalent behavior of each side of the diagonal line in these plots is axiomatic.

The ideal Flory bonding behavior in Figure 5a contrasts sharply with the results from our reactive Langevin simulations in Figure 5b, and the difference is quantified in Figure 5c. In the dynamical simulations, early stages of the reaction show increased formation of longer chains and reduced monomer–monomer bonding as compared to ideal behavior, a consequence of the aggregation spurred by chain lengthening. The propensity of longer chains to aggregate causes them to more readily encounter and react with longer chains and produces the length dependent increase to bonding, deviating from Flory’s ideal behavior. For the early stage of the reaction (*p* ≤ 0.4), these behaviors are largely independent of chain stiffness, as chain lengths at this stage are insufficient to drive the nematic ordering transition, which occurs only later in the reaction for stiff chains. As the reaction proceeds further (0.4 < *p* ≤ 0.7), the chain length dependence of bonding probabilities begins to differ between chains of different persistence length, as chains with sufficient stiffness begin to nematically align. Over this reaction extent range the difference is small, with flexible chains showing only a slight increase in the likelihood of bonding between longer chains. It is only in the late stage of the reaction (0.7 < *p* ≤ 0.9), when substantial nematic ordering has occurred, that greater differences in the bonding behaviors between flexible and stiff chains are observed. For flexible chains at this stage, there is an increased likelihood of reaction compared to the ideal Flory behavior for a wide range of oligomer chain length combinations. Flexible chains also have a particularly noticeable enrichment of bonding events that involve very long oligomers (length ≥50 monomers), as seen in the band of green along the top of the bottom plot in Figure 5c. By contrast, stiff chains show a marked increase of short chains (length ≤3 monomers) bonding with longer chains over this reaction extent, as visualized by the band of green along the *x*-axis. Both flexible and stiff chains also show a marked reduction in the bonding between shorter oligomers, visible in the purple region on the lower left. To further quantify these differences and how they develop as a function of reaction extent, in Figure S3 we report the relative proportion of bonding involving at least one chain of length ≥3 monomers and of bonding between both chains of length ≥3 monomers, for both flexible and stiff chains.

Taken together, the results in Figure 5 show that the transition to nematic ordering among the stiff chains, seen in the increase of the  metric near *p* = 0.4 in Figure 4c, occurs concomitantly with a change in chain-length dependent bonding behavior: a transition from longer chains preferentially reacting with longer chains to longer chains preferentially reacting with shorter chains. It is this shift in bonding behavior that results in the non-monotonic dispersity we see in Figure 4c. When this nematic ordering and the associated reduction in dispersity occur alongside the polymerization driven phase separation into **A**-rich and **B**-rich domains, peaked block length distributions emerge with a characteristic block length (Figure 4). Once chains have aligned, it becomes energetically favorable for oligomers shorter than the length of the growing crystallite to orient themselves such that their combined length spans the crystalline domain. Once in that position, a reaction between these aligned oligomers becomes more likely. We term this class of kinetics self-templating growth, and examine it in the context of the bonding behaviors of nucleated oligomers in the following section.

### 3.4 Oligomer Aggregate Structures and Bonding Behaviors Depend on Chain Stiffness

#### Quantitative Identification of Oligomer Aggregates

In order to examine this self-templating growth further, we now consider the initial aggregation, alignment, and growth of individual crystallites in the system. To this end, we first develop a consistent method of identifying aggregated regions in which the transition to nematic order and self-templated growth may occur. We therefore make use of a modification of the HDBSCAN clustering algorithm,^36^ which identifies spatially distinct oligomer clusters based on a local density criteria (see Sec. S4 in the SI for details). Clusters are initially identified as regions with a minimum of 12 monomers within a distance of 2.5σ. In addition, when one or more monomers of a chain are identified as belonging to a cluster, the entire chain is then added to that cluster. These conditions for cluster identification were chosen so that both small nucleated regions forming early in the reaction and larger, spatially separated aggregates occurring later in the reaction can be identified via consistent clustering criteria.

#### Aggregate Growth and Composition

**Figure 6:**
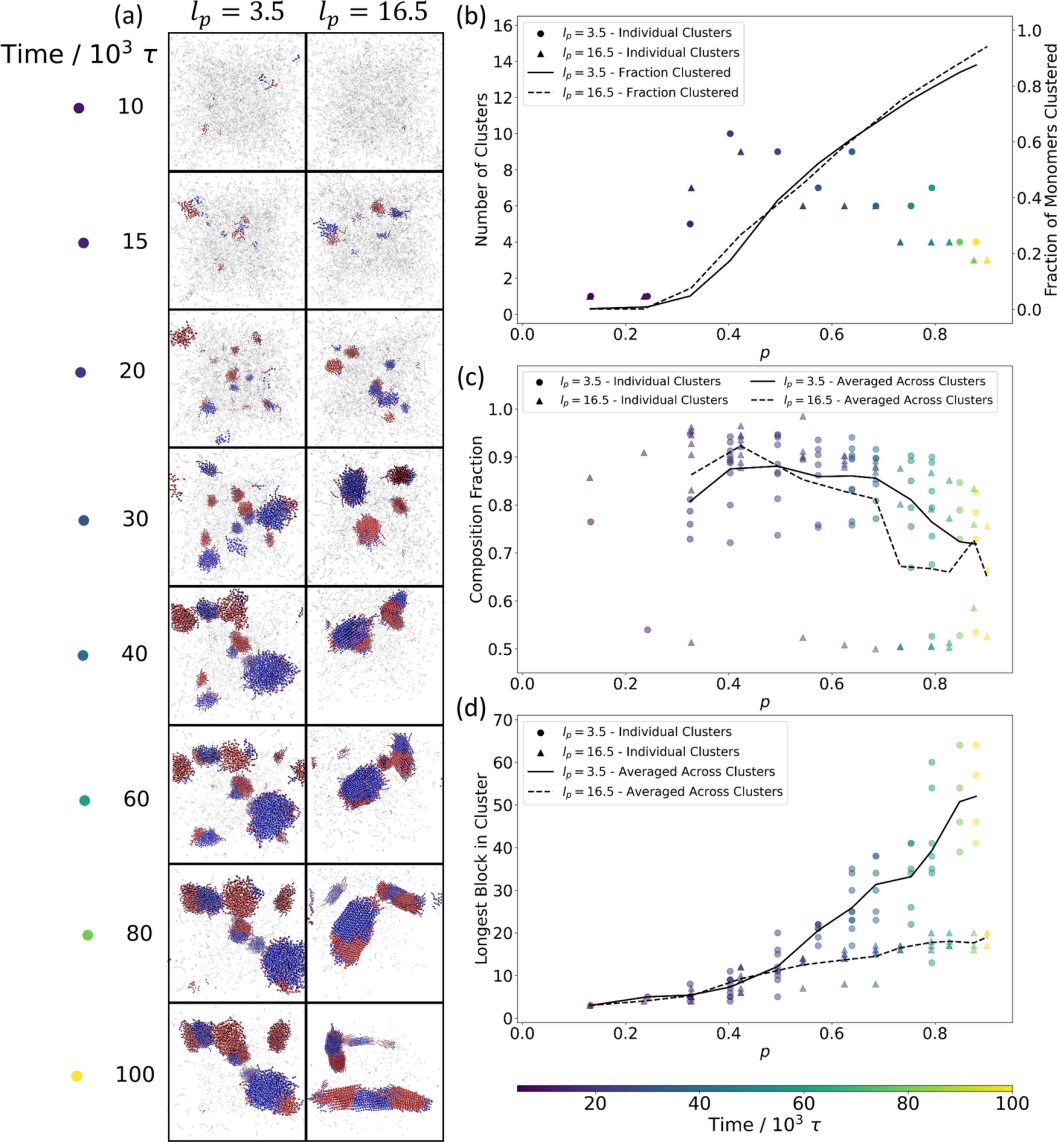
Identification of spatially clustered aggregates and their growth. (a) Aggregated structures identified by clustering analysis for both flexible (*l*_*p*_ = 3.5) and stiff (*l*_*p*_ = 16.5) chains. Monomers belonging to identified clusters are shown in color, with species **A** in red and species **B** in blue, while all other monomers are shown in transparent gray. (b) The number of separate clusters (points, left axis) and the fraction of all monomers belonging to a cluster (lines, right axis) are plotted vs the reaction extent, *p*. (c) The composition fraction of the dominant monomer species within a cluster is plotted as a function of the reaction extent, *p*. Each point corresponds to a single cluster identified at that reaction extent. Lines show the average value over all identified clusters. (d) The longest block length among all the sequences belonging to a single cluster is plotted here as a function of reaction extent, *p*. Each point corresponds to a single identified cluster at that reaction extent. Lines show the average longest block value over all of the identified clusters. In (b–d), marker coloration indicates simulation time, while marker style indicates chain stiffness.

The results of the density-based clustering analysis are presented in Figure 6. In Figure 6a, the growth of clusters identified within the simulation is visualized at different points throughout the reaction for both stiff and flexible chains. Irrespective of chain stiffness, no clusters are identified early in the simulation. Only at times ≥10 × 10^3^τ, where sufficient polymerization has occurred to drive assembly, do the first small clusters form. From 15 × 10^3^τ to 20 × 10^3^τ (*p* ≈ 0.3 to *p* ≈ 0.4) the number of identified clusters increases rapidly as the reaction continues and multiple oligomer clusters nucleate. Further aggregation proceeds through two pathways, with aggregates growing both from the addition of unaggregated chains from the surrounding, dilute phase and from the merging of separately nucleated clusters. This process of aggregate nucleation and subsequent growth and merger is quantified in Figure 6b, in which the number of distinct aggregates, indicated by the points, increases sharply during the initial nucleation phase before steadily decreasing throughout the remainder of the reaction as aggregates merge. Meanwhile, the total proportion of all monomers belonging to an aggregate increases continuously as monomers and chains not belonging to aggregates react with and join existing aggregates. Notably, this behavior appears to be independent of persistence length, as both flexible and stiff chains show nearly identical aggregate nucleation, growth, and cluster merging dynamics.

In Figure 6c, we consider the fractional comonomer composition of the clusters throughout the reaction. Due to the influence of the nonbonded attractions between like-monomer species, almost all aggregates are initially dominated by one comonomer species (Figure 6c). Of course, due to the relatively small size of clusters in the early stages of formation, some outliers in cluster composition occur early on where the comparatively rare inclusion of few monomers of the nondominant species in a cluster will have a disproportionate impact on the cluster composition as a whole. As the reaction continues and mergers between **A**-rich and **B**-rich aggregates become more frequent, the composition fraction starts to even out.

As seen in prior work^26,27^ and discussed in Section 3.1, the combination of local density increase and composition shift leads to a shift toward longer blocks of contiguous A or B sequences than would be present in a copolymerization which remains homogeneous throughout the reaction. This phenomenon is borne out within the individual aggregates, in which the longest block lengths in the aggregate (plotted for each cluster in Figure 6d) correspond to the largest block lengths observed in the system as a whole (Figure 4b), much longer than those of the unaggregated sequences in the system. Block lengths of unaggregated chains remain ≤3 monomers in length throughout the reaction (see Figure S6 in the SI), confirming that the increased block lengths observed in the system are isolated to the aggregated regions we identify. Although the initial nucleation, growth, and composition of aggregates are largely independent of chain stiffness (Figure 6b and c), the growth of contiguous sequence blocks within the aggregates differs between chains of different persistence lengths (Figure 6d).

#### Chain Stiffness Influences Bonding within and between Aggregates

In order to better understand this apparent contrast between similar aggregate growth pathways and dissimilar sequence development, we now consider the source of additions to growing aggregates. To this end, we consider three distinct populations of bond formation events for a given aggregate: (1) “unclustered” bonds, where a bond forms between a chain in the aggregate and a chain that is not part of any aggregate; (2) “self” bonds, where a bond forms between two chains that have been part of the same aggregate for at least 5 × 10^3^τ; and (3) “merge” bonds, where a bond forms between two chains that are part of separate, distinct aggregates.

**Figure 7:**
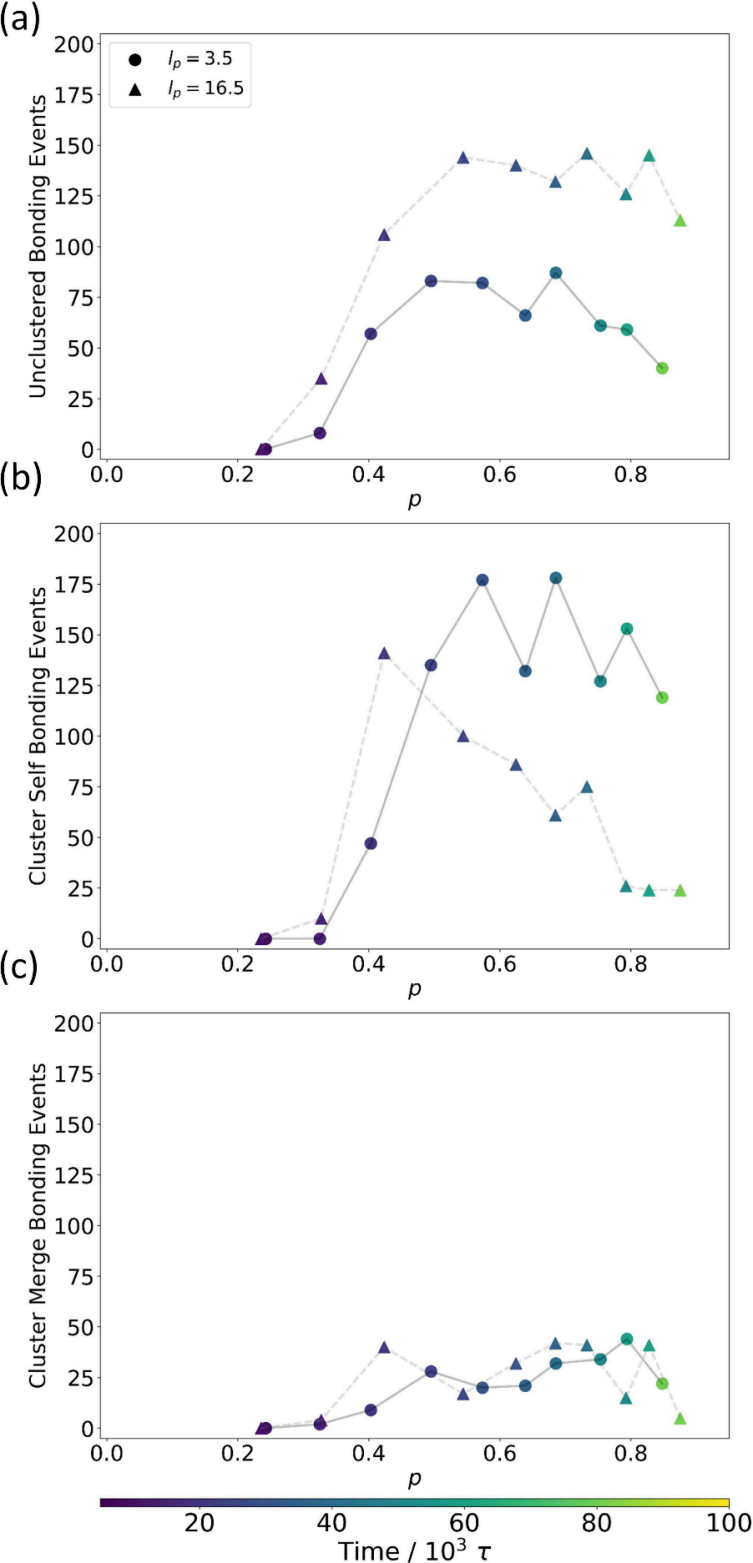
Oligomer bonding in spatially clustered aggregates. The number of oligomer bonding events occurring within aggregates identified by clustering analysis as a function of reaction extent for both flexible (*l*_*p*_ = 3.5) and stiff (*l*_*p*_ = 16.5) chains. Bonding events were split into three populations: (a) bonding with unclustered reactants, (b) bonding within a single cluster, and (c) bonding between separate clusters. Each data point corresponds to the total number of bonding events of each type among all clusters in the system to the indicated reaction extent. Time is indicated by marker coloration, and chain stiffness is indicated by marker style. Lines connecting data points are included for eye guidance only. Solid lines provide this guidance for flexible chain data points, and dashed lines provide it for stiff chain data points.

Figure 7 shows the results of this bonding population analysis, performed on the same flexible and stiff chain systems as shown in Figure 6. One of the most readily observable differences in bonding behavior between flexible and stiff chain aggregates lies in the number of unclustered bonding events (Figure 7a), as the stiff chain aggregates form more bonds with the unclustered population of chains than the flexible chain aggregates form. This change in bonding behavior is critical: unclustered chains have shorter chain and block lengths than those in aggregated regions (Figures S5 and S6), since they have not been influenced by the emerging phase-separation and assembly effects. The unclustered chains with shorter than average chain and block lengths bond with aggregated chains with longer than average chain and block lengths, narrowing the respective distributions toward the mean. Increased incorporation of these unclustered chains in the stiff chain case therefore serves to reduce the dispersity of both chain and block lengths and slows the distribution broadening caused by aggregation. For bonding occurring between chains within the same cluster (Figure 7b), the opposite relationship with persistence length is observed. Here, stiff chain aggregates demonstrate reduced self-bonding behavior as compared to flexible chain aggregates after the transition to nematic ordering has begun (*p* > 0.4). Thus, for flexible chains, bond formation continues to occur preferentially between chains within the aggregated population. Due to the longer chain and block lengths of that population, this propensity toward self-bonding causes chain length and block length dispersities to continue to increase. Notably, this difference in bonding behaviors of aggregates of different chain stiffness is not seen in bonding events associated with the merger of separate clusters (Figure 7c). Bonds from merging clusters make up a smaller portion of the total bonding events observed, and the quantities of these events and the timing of their occurrence are relatively consistent between both stiff and flexible chains.

#### Aggregate Structure Alters Accessibility of Reactive Chain Ends

In considering the impact of chain stiffness on bonding within and between aggregates, we hypothesized that both of the above observations of altered aggregate bonding behavior are related to the relative positioning of chain ends within the aggregate structures. In flexible chains and in stiff chains prior to nematic ordering, chain ends may be isolated in the interior of an aggregate, where they are less readily accessible to chains that are not a part of the same aggregate. In contrast, the ordering transition that stiff chain aggregates undergo positions chain ends on the exterior of the aggregated structure. This positioning not only increases the accessibility of chain ends to the local environment surrounding the aggregate but also limits contact between the reactive chain ends of inflexible chains within the same aggregate, simultaneously promoting bonding from outside the aggregate and limiting bonding from within it. The timing of this ordering transition for stiff chains is therefore critical, as the initial aggregation promotes distribution broadening by increasing bonding between the longer chains within an aggregate, and then nematic alignment promotes bonding with shorter chains from the surrounding environment.

**Figure 8:**
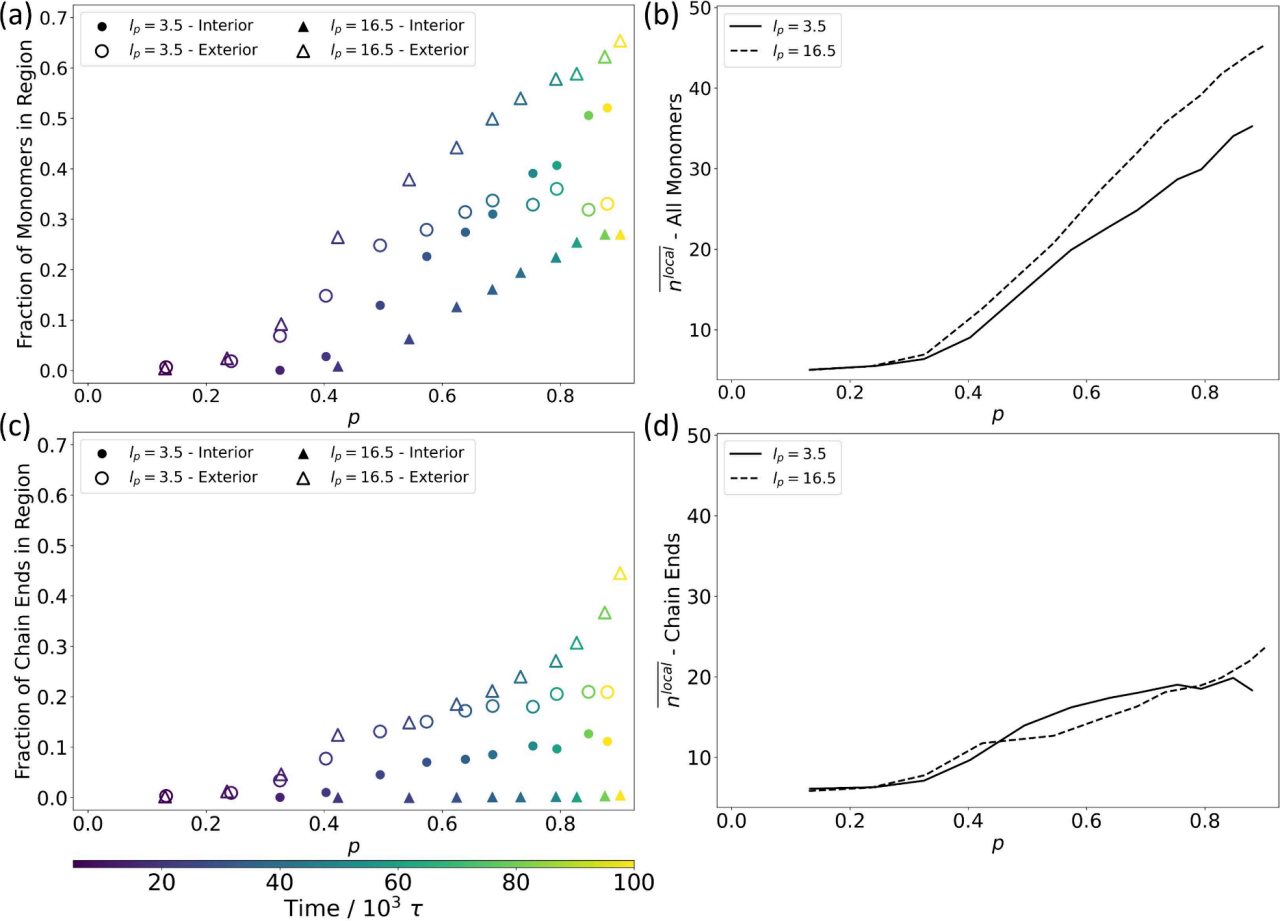
Distribution of chain ends within aggregates. The fractions of (a) all monomers and (c) all chain end monomers in the system that belong to an interior or exterior region of an aggregate are plotted as a function of the reaction extent, *p*, for both flexible (*l*_*p*_ = 3.5) and stiff (*l*_*p*_ = 16.5) chains. The average number of local neighbors, , for (b) all monomers and (d) all chain end monomers in the system, is plotted vs reaction extent for flexible and stiff chains. Aggregate regions were identified by the local density criteria using *n*^local^ to quantify the number of neighboring monomers within 2.5σ; monomers with *n*^local^ ≥ 36 were identified as interior, while monomers with 12 ≤ *n*^local^ < 36 were identified as exterior.

In Figure 8, we quantify the spatial distribution and local reactant density of all monomer units and of all chain ends within aggregates of both stiff and flexible chains. When considering all aggregated monomer units, both free monomers and those bound within oligomer chains, in Figure 8a, we see that the fractions of all monomer units in the simulation belonging to the interior or exterior of an aggregate both increase with reaction extent. The largely spherical aggregates produced by flexible chains have more of their aggregated monomers in the interior regions, as compared to the more extended aggregates of stiff chains, which have more of their aggregated monomers in the exterior regions. The average number of local (within 2.5σ) neighbors, *n*^local^, in the system is plotted in Figure 8b for all monomers. As expected, the local density increases monotonically with reaction extent due to the polymerization driven by aggregation. Despite their more extended character, however, the stiff chains demonstrate a higher average density after nematically ordering, a consequence of the closely packed structures such alignment produces.

When considering the fraction of chain end monomers in aggregate exteriors and interiors in Figure 8c, we find that chain ends are found exclusively in the exteriors of stiff chain aggregates. In contrast, an appreciable number of chain ends remain in the interior region of flexible aggregates, and this number grows steadily as the reaction proceeds and the interior volume increases. These differences can also be seen in the *n*^local^ of the chain ends in Figure 8d. Although the curves initially look the same as those for all monomers (Figure 8b), a crossover occurs at *p* ≈ 0.4, when the stiff chains begin to nematically order, after which the flexible chain ends show greater neighbor density than their stiff chain counterparts. Only very late in the reaction, *p* ≳ 0.8, does the local density near the stiff chain ends again surpass those of flexible chains.

Overall, the nematic ordering in stiff chains ensures that chain ends, where new bond formation can occur, are restricted to the less dense exterior regions of aggregates (Figure 8c and d) where they are more readily accessible for bond formation with shorter, unaggregated chains (Figure 7, Figure S5 in the SI).

## 4 Conclusion

In this work we have investigated a previously reported^30^ templating effect in step-growth copolymerizations of monomer species with sufficient chain stiffness and nonbonded interactions between like species, an effect which results in a characteristic block length of sequence repeats. Here, we have examined the response of this characteristic block length to changes in reaction conditions, demonstrating its sensitivity to changes to the relative time scales of diffusion and reaction, such as those introduced by changes to solution viscosity or reaction activation energy. We have shown that the characteristic block length shifts in response to changes in these time scales in a quantifiable fashion, suggesting a pathway for biasing copolymer sequences toward a particular block length through understanding this effect.

Exploring the development of the templating effect in time shows that the emergence of the characteristic length coincides with a phase separation and nematic ordering in the system unique to stiff chains and that this drives changes in length dependent bonding behavior associated with these transitions. It is the simultaneous impact of these effects that together produce the unique characteristic block length behavior we observe. These results further contextualize and reinforce the impact of characteristic system time scales; because the characteristic block length develops as a consequence of both phase separation and nematic ordering, changes to the relative time scales of diffusion and reaction, which govern the rates of these processes, necessarily alter the point at which self-templating growth begins, the extent to which it influences sequences, and thereby the characteristic block length that emerges.

To further understand how the combination of nematic ordering and phase separation introduces length dependent bonding behaviors in stiff chain oligomers, we have contrasted the growth pathways of aggregated oligomer structures of flexible and stiff chains. We have highlighted the differences in bonding behaviors of these aggregates in terms of the reactant populations that spur their growth and motivated this in terms of the relative accessibility of unreacted chain ends to these populations. Together, these results explain the unique chain stiffness and chain length dependence of bonding we observe and support our description of the impact of characteristic system time scales on the development of a characteristic block length. The structure of aggregates and their growth over time are what are fundamentally altered by nematic ordering, and it is this change, in concert with the ongoing phase separation in the system, that leads to the unique bonding behaviors we observe. Changes to characteristic time scales in the system alter when these aggregates form and when they order, relative to the rate at which polymerization proceeds and sequences and chain lengths develop, promoting or truncating the impacts of aggregation and ordering on these features.

Our results have demonstrated how the templating effect arises from the simultaneous impacts of polymerization-driven phase separation and nematic transition, which alter the bonding behavior of nascent chains in a length-dependent fashion. These length-dependent bonding behaviors depend on the formation, growth, and structural ordering of oligomer aggregates, which change the accessibility of the aggregated oligomers to other populations of reactants. Altering the characteristic reactive and diffusive time scales in the system changes the timing of formation of aggregates and their ordering with respect to the progress of the reaction, changing the extent to which the chain-length and chain-stiffness dependent bonding behaviors may influence sequence development, and thereby shifting the resultant characteristic sequence development that emerges.

It should be noted that this work has made use of simplifying assumptions with respect to the activation energy and persistence length of the reactants by setting equivalent values for each of the comonomers and their pairs within the simulations. While such simplifying assumptions allow for a more direct examination of the sequence templating behaviors by excluding these confounding variables, real world polymerizations frequently have notable differences in reactivity or chain stiffness between the polymerizing species, and such variations would need to be considered for any specific copolymerization reaction. Previous works by the authors and co-workers have also explored the impacts of asymmetries in these specific variables on this model copolymerization system,^28,30,37^ finding that, while the specific interplay between these variables and polymerization induced phase separations is complex, the underlying phenomenon observed in this work persists even when chain stiffness or reactivities vary.

Previous work has posited similar length matching effects in aligned polymers with nonbonded attractions between neighboring chains, and we observe such a cooperative aggregation effect in our own system, which biases our nematically ordered aggregates toward structures with fewer “loose ends”,^38^ i.e., toward structures in which the lengths of neighboring chains match. Though distinct in mechanism, the “gel effect”^39,40^ also produces autocatalytic kinetics and length dependent bonding behaviors^41^ in radical polymerizations analogous to the behaviors observed in this work, through emergent and length-dependent changes to the diffusion of reactants. Specifically, the reduced diffusion of longer chains slows the rate of termination, favoring the competing propagation step and thereby increasing the overall polymerization rate. By contrast, in our system, autoacceleration and length dependent bonding arise instead from nonbonded interactions and the associated aggregation, demixing, and nematic ordering of chains of sufficient length and stiffness. In the subsequent growth of the aggregated phase, templating behavior occurs, and the characteristic block length emerges.

The results presented in this work are of particular interest in the context of rod–rod conjugated copolymers,^42,43^ in which both comonomer species exhibit backbone rigidities analogous to the stiff chain copolymers explored here.^44^ Rod–rod conjugated copolymers have demonstrated a number of optoelectronic material properties of interest,^42,45−47^ and crucially, these properties have been shown to be strongly influenced by the primary monomer sequence and blockiness.^48−50^ The collective development of characteristic sequence repeats we observe here provides a potential avenue for directly biasing copolymer sequence in a wide range of chemical systems through the informed modification of the balance of reactive and diffusive time scales of the reaction. Further, the behavior of oligomeric aggregates of conjugated polymers has attracted attention as a way to both improve solvent processability and tune material properties through intentional exploitation of mesoscale structuring.^51−54^

Our results provide insight into the development of oligomeric aggregates in stiff chain copolymer systems, showcasing the interplay between aggregate structure and ordering and the resulting sequences and molecular weight distributions of growing oligomers.

## Supporting Information Available

Simulation details, chain length dependence of bonding statistics, block length statistics, clustering analysis, system parameters and characteristic time scale, and characteristic length in homopolymer system (PDF)
