# Supplementary material for: Characteristic System Time Scales Can Influence the Collective Sequence Development of Nematically Ordered Copolymers
Source: Macromolecules. 2024 Oct 15;57(21):9984–98. doi: 10.1021/acs.macromol.4c01047 (PMC11562797; doi:10.1021/acs.macromol.4c01047)
Supplement: Supplementary file 1 — ma4c01047_si_001.pdf [file ma4c01047_si_001.pdf]

# Supporting Information: "Characteristic System Timescales Can Influence the Collective Sequence Development of Nematically Ordered Copolymers"

Ryan L. Hamblin,† Zhongmin Zhang,‡ and Kateri H. DuBay*,†

†*Department of Chemistry, University of Virginia, Charlottesville, VA*
‡*Department of Chemistry, University of North Carolina at Chapel Hill, Chapel Hill, NC*

E-mail: dubay@virginia.edu

## S1 Simulation Details

The original model we use here was described and discussed in detail in Ref. S1&S2. Details specific to this work are described below. All simulations were performed in LAMMPS.[S3]

### S1.1 Non-bonded interactions

Intermolecular interactions between central "type-**1**" particles are modelled via a modified Lennard-Jones potential, defined as:

$$E_{LJ}(1,1') = \begin{cases} 4\varepsilon_{\mathrm{att}(1,1')}[(\frac{\sigma}{r_{(1,1')}})^{12} - (\frac{\sigma}{r_{(1,1')}})^{6}] & r_0 \le r_{(1,1')} < 2.5\sigma \\ 4\varepsilon_{\mathrm{rep}(1,1')}[(\frac{\sigma}{r_{(1,1')}})^{12} - (\frac{\sigma}{r_{(1,1')}})^{6}] + c & r_{1,1'} < r_0, \end{cases} \tag{S1}$$

in which $r_0$ is the equilibrium separation distance at which the potential reaches a minimum, $r_{1,1'}$ is the distance between two type-**1** particles, and $\varepsilon_{\mathrm{rep}}$ is a constant which controls the repulsion between central particles and is kept fixed for all monomer species pairs. Similarly, $\varepsilon_{\mathrm{att}}$ controls the well-depth of the attractive portion of the potential, and is assigned based on monomer identity, i.e., $\varepsilon_{AA}$, $\varepsilon_{BB}$, and $\varepsilon_{AB}$ for **A**:**A**, **B**:**B**, and **A**:**B** attractions, respectively. The constant $c$ is set to $c = \varepsilon_{\mathrm{rep}}$ - $\varepsilon_{\mathrm{att}}$ to ensure continuity between the attractive and repulsive portions of the potential. The two-part nature of the potential allows us to maintain fixed repulsive forces between all monomer, while simultaneously adjusting the strength of attractions by monomer species.

In order to model a reactive process with an adjustable activation energy, we include a soft, short-ranged and purely repulsive potential between external "type-**2**" particles via:

$$E^{2,2'}_{\mathrm{soft}} = \begin{cases} \frac{E^{ij}_{\mathrm{barr}}}{2}\cos\frac{\pi(r_{2,2'}-d_{\mathrm{bond}})}{d_{\mathrm{on}}-d_{\mathrm{bond}}} + \frac{E^{ij}_{\mathrm{barr}}}{2} & d_{\mathrm{bond}} < r_{2,2'} < d_{\mathrm{on}} \\ 0 & r_{2,2'} \ge d_{\mathrm{on}} \end{cases} \tag{S2}$$

in which $d_{\mathrm{on}}$ is the cutoff distance for the potential and $E^{ij}_{\mathrm{barr}}$ is the height of this contribution to the reaction barrier, which can be adjusted across different $ij$ monomer pairs.

### S1.2 Angular potential.

Intramolecular and intermolecular angles are governed via the harmonic potential:

$$E_{\mathrm{angle}}(\theta_{ijk}) = K^{\mathrm{angle}}_{ijk}(\theta_{ijk} - \theta_0)^2,$$

in which $\theta_{ijk}$ is the angle between particles $i$, $j$ and $k$, $\theta$ is the equilibrium angle and $K^{\mathrm{angle}}_{ijk}$ is the spring constant for the angle. For the intramonomer angle between particle **2-1-2′**, $\theta_0$ = 180° and the spring constant is set to $K^{\mathrm{angle}}_{212'}$ =

$5\,\varepsilon$ rad$^{-1}$ and $K^{\text{angle}}_{212'} = 50\,\varepsilon$ rad$^{-1}$ for the flexible chains and stiff chains, respectively. For the intermonomer angle, **1-2-2′**, $\theta_0 = 180°$, $K^{\text{angle}}_{212'} = 100\,\varepsilon$ rad$^{-1}$ for both flexible chains and stiff chains.

### S1.3 Langevin dynamics and viscosity

Langevin dynamics was implemented in LAMMPS using the "fix langevin" command. A *damp* parameter is used to control the diffusion rate and the relaxation rate of the temperature, which is discussed in detail in the Supplemental Information of Ref. S2. The values of *damp* and the associated viscosities explored in this work are shown in Table S1. In the "standard" simulations in this work (marked with a * in Table S1, the *damp* parameter is set to 0.1 $\tau$, resulting in a viscosity of $\gamma = 0.1$ mPa $\cdot$ s. For further discussion of this parameter, see Ref. S2.

**Table S1: Simulation viscosity.** The first column shows the value of the Langevin parameter, *damp*, and the second the associated viscosity in units of mPa·s. The "standard" conditions are marked with a *.

| *damp* ($\tau$) | Viscosity (mPa·s) |
|---|---|
| 0.01 | 1.00 |
| 0.03 | 0.3 |
| 0.05 | 0.2 |
| 0.1* | 0.1* |
| 0.3 | 0.03 |
| 0.5 | 0.02 |
| 1.0 | 0.01 |

### S1.4 Activation energies

To ensure that having a fixed value for $\varepsilon_{\text{rep}}$, plus the additional soft repulsive potential between type **2** particles, was maintaining a consistent activation energy across monomer type pairs for our range of simulation parameters, we tabulated the activation energies for successful bond formations for a set of ~60000 bonding events drawn from the full range of simulation parameters explored in this work. Activation energies are calculated from the total potential energy of the two bonding monomers arising from the LJ potential between type **1** particles, the soft repulsive potential between type **2** particles, and the intramonomer bond lengths and angles. The values of each of these contributions above their minimum energy are summed at the time of bond formation to obtain the total activation energy for the bonding event. Fig. S1 shows the results of this comparison, broken down by monomer type pairs and attraction strength combination, demonstrating consistent activation energies across monomer type pairing and simulation parameters.

### S1.5 Persistence lengths.

Persistence length determination was previously presented in Ref. S2. Single chains of purely repulsive 100mers at several chain stiffnesses were simulated in order to calculate the persistence length, $l_{\text{p}}$, that corresponds to each value of $K^{\text{angle}}_{212}$. The value of $l_{\text{p}}$ in each case was calculated using the relation $\langle \cos\theta \rangle = \exp(-l/l_{\text{p}})$. Here, $l$ is the contour length distance between two points on the polymer chain, and $\theta$ is the angle between the tangent lines drawn at each of these two points. Persistence lengths are tabulated in monomer units, $l{=}\Delta n$, where $n$ is simply the ordinal number of each monomer in the chain. An average is taken over an equilibrium ensemble of configurations at each contour length distance of $l$.

## S2 Chain length dependence of bonding statistics.

The Flory-Schulz distribution[S4] is a well-studied and validated distribution which describes the probability, $P(x)$ of obtaining chains of length $x$ in a step-growth polymerization reaction in terms of the reaction extent, $p$, as:

$$P(x) = (1-p)p^{x-1}. \tag{S3}$$

The fundamental simplifying assumption at

the heart of this expression is the so-called "Flory's equal reactivity principle" which supposes that the likelihood of reaction between two polymerizing species is completely independent of the chain length of either reactant. Though Flory himself acknowledged that this principle would breakdown under heterogeneous reaction conditions,[S4] such as those caused by the emergent demixing in the system studied in this work, this assumption serves as a worthwhile starting point for the purposes of highlighting the chain-length dependence in bonding which we observe.

In order to translate this assumption into probabilities of particular chain length pairs forming a bond at each stage of the reaction, allowing us to thereby compare our simulation results to those expected from the equal reactivity case, we performed a series of simple Monte Carlo simulations. Each simulation begins with a collection of 7200 monomers, just as in the full Langevin dynamics simulation of our system. We then choose two monomers, with uniform probability, and cause them to react. This process is performed iteratively, maintaining a uniform selection probability for all reactants, until a reaction extent of $p = 0.9$ is reached. This was repeated over a series of 250 Monte Carlo simulation trials. The resulting bonding events were tabulated and split into three stages of the reaction $p \leq 0.3$, $0.3 < p \leq 0.6$, and $0.6 < p \leq 0.9$, corresponding to the early, middle, and late stages of the reaction respectively. The histogram of the resulting bonding events is provided in Fig. 5a in the main text. The chain length distribution we obtain from this Monte Carlo simulation process matches the Flory-Schulz prediction from Eq. S3 nearly identically, as shown in Fig. S2 below. We note here that this precise matching of Flory-Schulz predictions is also seen in Langevin dynamics simulations of our full model under conditions with sufficiently weak non-bonded attractions, as reported in our previous works.[S1,S5,S6]

The chain-length dependent bonding behavior and its sensitivity to chain stiffness, discussed in Fig. 5 in the main text, is further demonstrated in Fig. S3. Fig. S3 shows the proportion of new bond formation involving

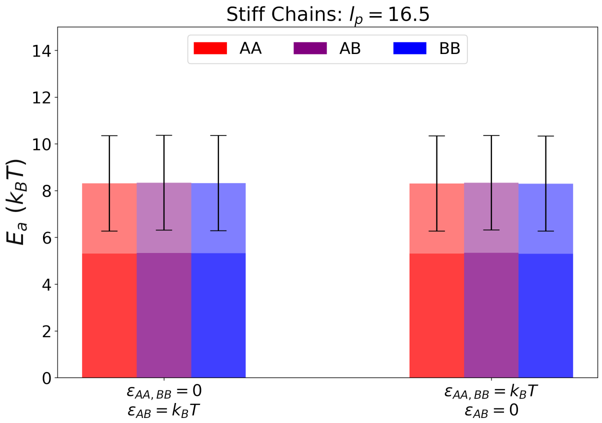


**Figure S1: Activation energies.** The activation energies, $E_a$, encountered during the polymerization reaction. Bonding events were sampled from simulations under standard conditions and are shown here for two attraction strength combinations: $\varepsilon_{AA,BB} = k_BT, \varepsilon_{AB} = 0$ and $\varepsilon_{AA,BB} = 0$ , $\varepsilon_{AB} = k_BT$). Sample populations are further separated by monomer pair, namely **AA**, **AB**, and **BB**. In total, ~60000 bonding events were sampled. Mean values of the measured activation energies for each combination are shown, with the error bars representing the standard deviation. The dark shades of colors represent the constant component of activation energy, composed of LJ repulsions and geometric constraints, which has an average value of $\sim 5.3 k_BT$. The lighter shaded region represents the short-ranged repulsion between type-2 particles from $E_{barr}$, which is varied across simulations to control the total activation energy.

oligomers, which we define to be chains of length $\geq 3$. Both the nematic ordering transition and the reduction in chain length dispersity are unique features of chains with sufficient stiffness. Flexible chains remain disordered throughout the reaction, and dispersity continues to increase monotonically. As such, while the fraction of oligomers participating in bond formation is similar between flexible and stiff chains (Fig. S3a), bond formation in which both of the reacting chains are oligomers are significantly less likely for stiff chains after nematic alignment occurs (Fig. S3b).

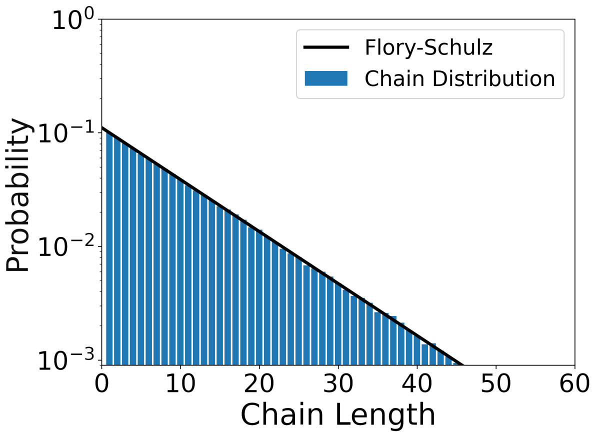


**Figure S2: Chain length distribution from Monte Carlo simulation of equal reactivity.** The observed chain length distribution at $p = 0.9$ for 250 Monte Carlo simulation trials with uniform reaction probability. The predicted distribution from Flory-Schulz theory is also shown.

# S3 Block Length statistics.

**Markovian block length statistics.** Under the assumption of Markovian statistics, where the probability of having a monomer type in the chain depends solely on the identity of the preceding monomer, the expected probability of having of a continuous block of length $n$ of repeats of **A** or **B** in the copolymer sequence can be calculated via:

$$P(n) = (1 - pp_{\mathrm{AA,BB}})(pp_{\mathrm{AA,BB}})^{\mathrm{n}-1},$$

where $p$ is the reaction extent and $p_{\mathrm{AA,BB}}$ is the

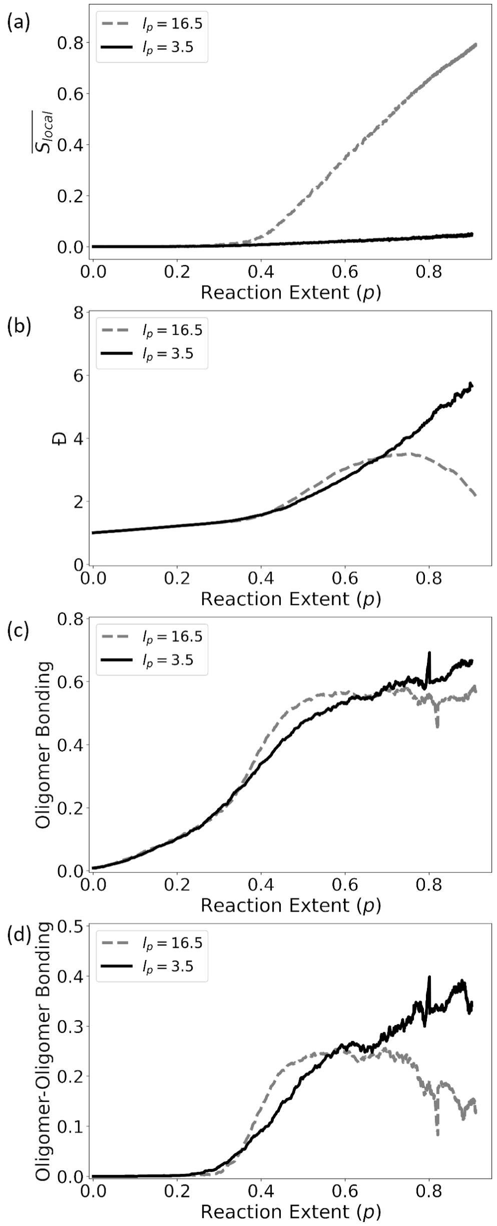


**Figure S3: Proportion of oligomers reacting.** (a) Local nematic ordering parameter, $\overline{S_{\mathrm{local}}}$, as a function of reaction extent. (b) Chain length dispersity, $Đ$, as a function of reaction extent. (c,d) The fractions of all bonding events occurring in which (c) one of the reacting chains is an oligomer and (d) both of the reacting chains are oligomers, are shown as a function of reaction extent $p$. Oligomers are defined as chains of three or more monomers. All results in (a-d) are shown for both flexible ($l_p = 3.5$) and stiff ($l_p = 16.5$) chains, and are obtained from three independent simulation trials for each chain stiffness.

probability of having a like nearest neighbor in the sequence.

**Quantifying deviations from Markovian predictions.** To quantitatively explore the deviation from Markov statistics in our system, we compared the Markovian predictions for dispersity and block length distribution with the results of our simulations. We calculated the Wasserstein metric,[S7,S8] $W_1$, as a measure of the statistical distance between the Markovian distributions and our observed block length distributions.

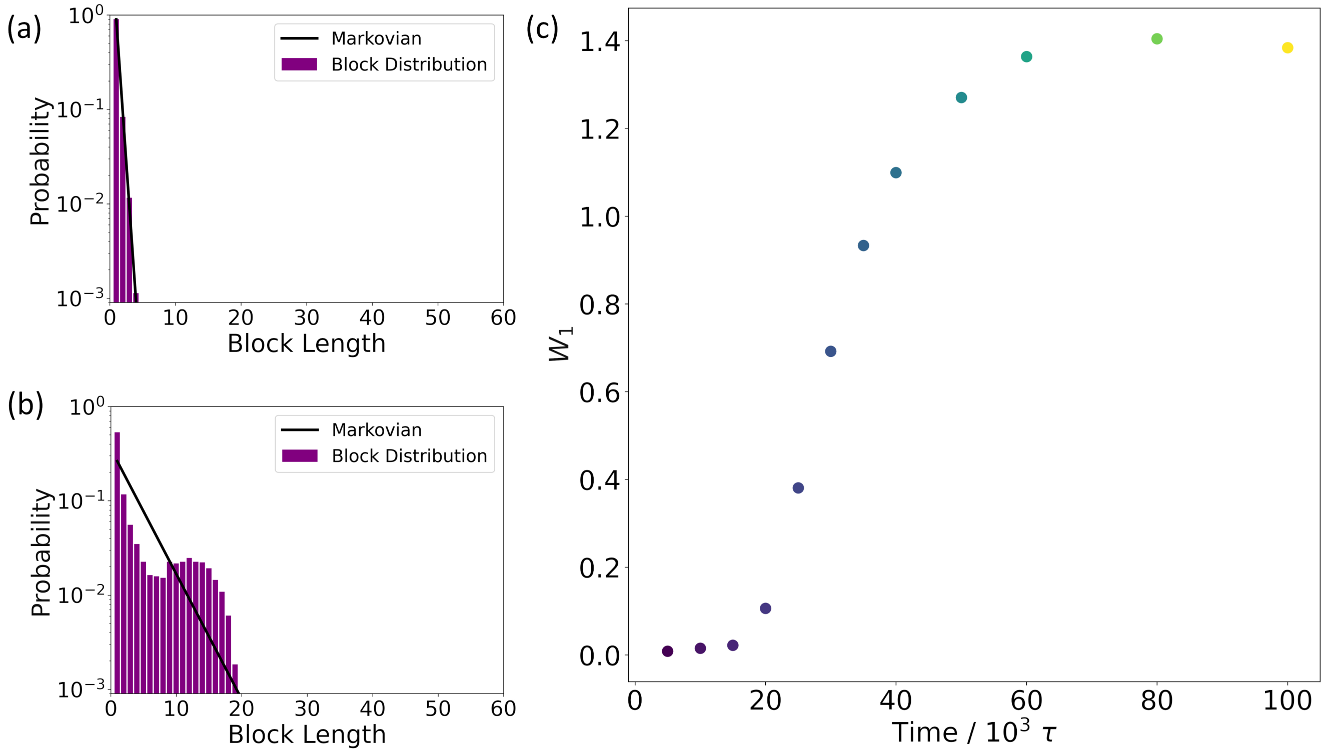


**Figure S4: Block distribution deviation from Markovian statistics in time.** The observed block length distribution (discussed in Fig. 4b in the main text) at (a) $5 \times 10^3 \tau$ and (b) $100 \times 10^3 \tau$ are plotted alongside the distribution expected from Markov statistics. (c) The Wasserstein distance, $W_1$, is calculated between the observed block length distribution and the Markovian distribution and plotted as function of time, for points corresponding to the block distributions shown in Fig. 4b in the main text.

## S4 Clustering Analysis

Identification of aggregates was performed via the use of the HDBSCAN[S9,S10] algorithm, which uses a density based criteria for cluster identification. At each time point chosen for analysis, HDBSCAN clustering was performed on the spatial coordinates of all monomer centers in the system, with a minimum cluster size of twelve monomers, chosen for consistency with our definition of $\overline{S_{\mathrm{local}}}$. To ensure copolymer chains had consistent cluster definition, all monomers within a chain where assigned to a cluster in which any member of the chain was assigned. When any given chain had monomers belonging to more than one cluster, those clusters were merged and all members of each cluster where assigned to the new, merged cluster.

Additionally, the distribution of chain lengths and block lengths for both flexible and stiff chains were calculated for all chains belonging to a cluster, and all chains which remained unclustered. These distributions are shown in Fig. S5&S6 below, alongside the Wasserstein distance between the observed and predicted distributions.

To explore spatial differences in **A**,**B**-interface formation between flexible and stiff chains, we again employed a density based clustering approach, this time identifying the extent of clustering of bond types, namely **AA**, **AB**, and **BB** bonds. The proportions of spatially clustered bonds by bond type are shown in Fig. S7, alongside the sequence neighbor probabilities, $p_{\mathrm{AA}}$, $p_{\mathrm{AB}}$, and $p_{\mathrm{BB}}$, *i.e.*, the proportion of **AA**, **AB**, and **BB** bonds respectively. Notably, in both flexible (Fig. S7a) and stiff (Fig. S7b) chains, the sequence neighbor probabilities, $p_{ij}$, behave nearly identically for $p > 0.2$, the range in which bond clustering begins and for which sufficient bonding has occurred for $p_{ij}$ values to be well sampled. Likewise, the proportion of **AA** and **BB** bonds which are clustered behaves similarly for both flexible and stiff chains, demonstrating nearly complete spatial clustering as a consequence of the emergent phase-separation. In stiff chains, however, **AB** bonds show an earlier emergence of clustering, concomitant with the ordering transition, which reaches a significantly higher extent than that seen for flexible chains. This is a clear indication of the formation of densely co-located **AB** interfaces created when nematically ordered **A**-rich and **B**-rich aggregates merge.

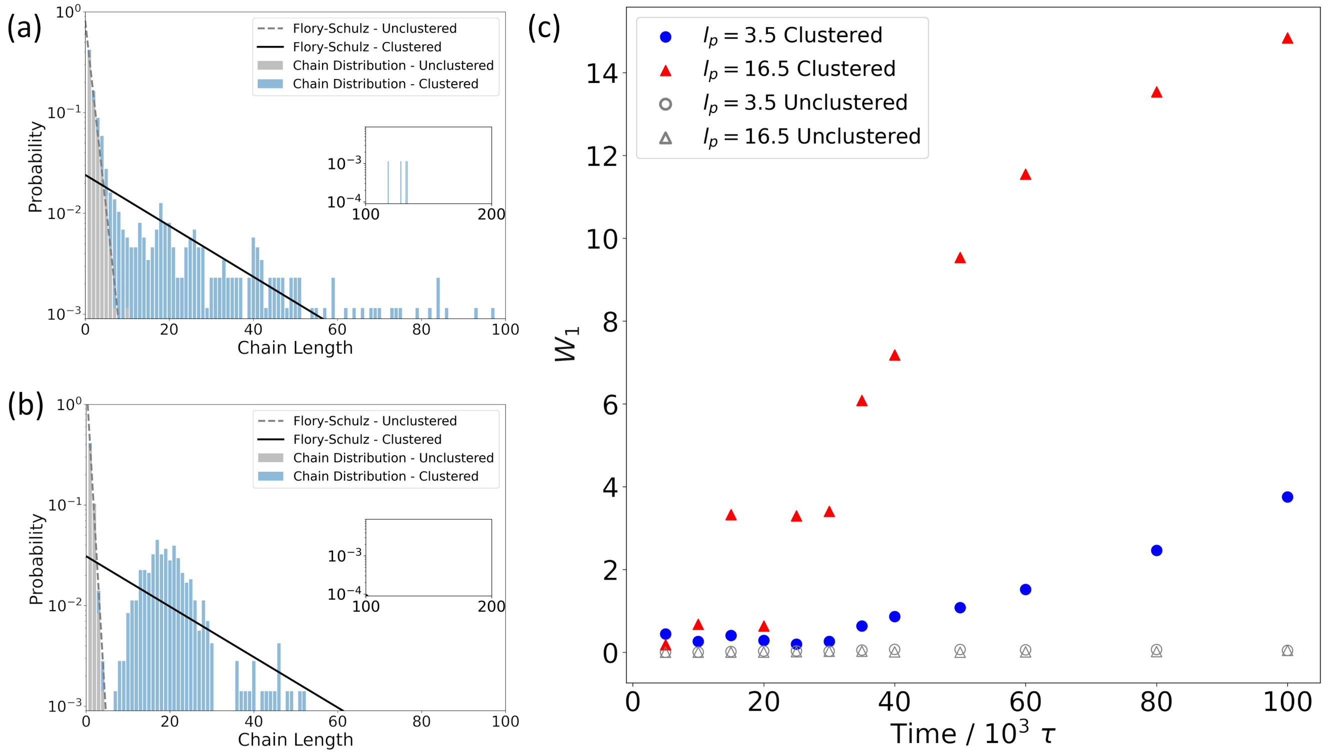


**Figure S5: Chain distribution statistics for clustered and unclustered populations.** The observed chain length distribution at $p = 0.9$ for (a) flexible chains ($l_p = 3.5$) and (b) stiff chains ($l_p = 16.5$) are plotted alongside the Flory-Schulz distribution. Chains were separated into clustered and unclustered populations based on the clustering analysis described above, with a corresponding reaction extent, $p$, within each population. The observed chain distribution is separated into clustered (blue) and unclustered (gray) portions, with the associated Flory-Schulz prediction obtained from the specific reaction extent of the population. (c) The Wasserstein distance, $W_1$, is calculated between the observed chain length distribution and the Flory-Schulz distribution and plotted as function of time, for points corresponding to the block distributions shown in Fig. 4b in the main text. Results for both clustered and unclustered populations of both flexible ($l_p = 3.5$) and stiff ($l_p = 16.5$) chains are shown, as indicated by marker coloration and style.

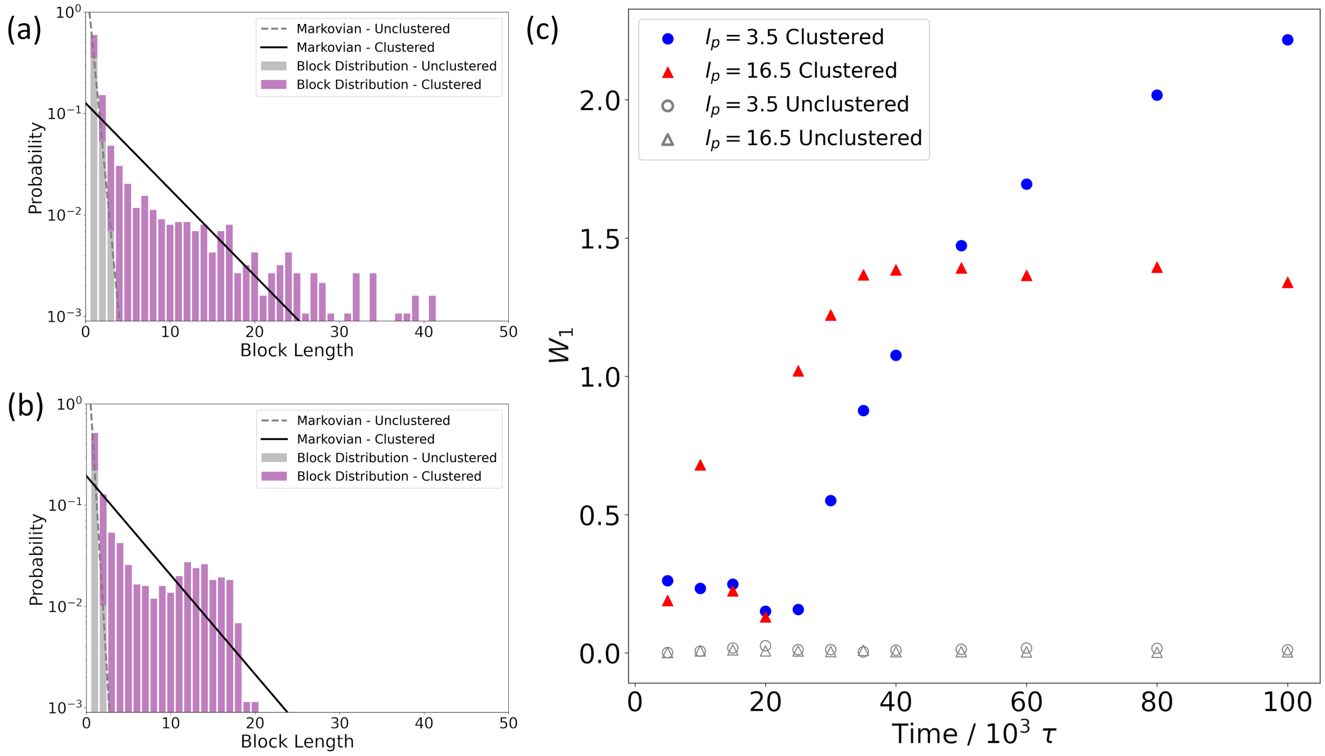


**Figure S6: Block distribution statistics for clustered and unclustered populations.** The observed block length distribution at $p = 0.9$ for (a) flexible chains ($l_p = 3.5$) and (b) stiff chains ($l_p = 16.5$) are plotted alongside the distribution expected from Markov statistics. Sequences were separated into clustered and unclustered populations based on the clustering analysis described above, with a corresponding reaction extent, $p$, and value of $p_{\text{AA,BB}}$ within each population. The observed block distribution is separated into clustered (purple) and unclustered (gray) portions, with the associated Markovian prediction obtained from the specific reaction extent and $p_{\text{AA,BB}}$ of the population. (c) The Wasserstein distance, $W_1$, is calculated between the observed chain length distribution and the Markovian distribution and plotted as function of time, for points corresponding to the block distributions shown in Fig. 4b in the main text. Results for both clustered and unclustered populations of both flexible ($l_p = 3.5$) and stiff ($l_p = 16.5$) chains are shown, as indicated by marker coloration and style.

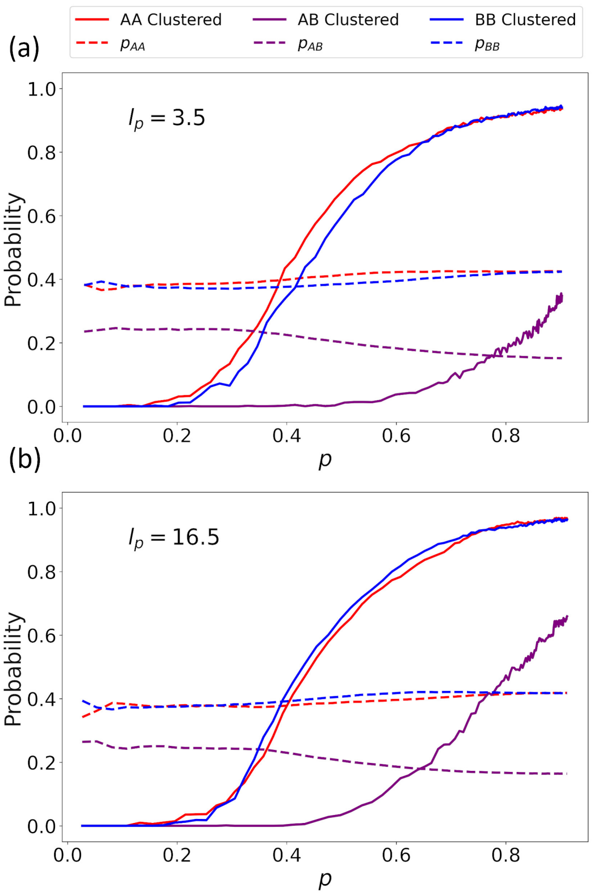


**Figure S7: Spatial clustering of bonds by type.** Fractions of bonds formed that satisfy clustering criteria as a function of reaction extent for (a) flexible chains ($l_p = 3.5$) and (b) stiff chains ($l_p = 16.5$). Bonds were identified as clustered if there were at least 6 other bonds of the same type within $2.5\sigma$. Also shown are the sequence neighbor probabilities, $p_{\mathrm{AA}}$, $p_{\mathrm{AB}}$, and $p_{\mathrm{BB}}$: respectively, the probabilities of observing an **AA**, **AB**, or **BB** pair within all sequences in the system. Coloration indicates the bond type: red for **AA**, purple for **AB**, and blue for **BB**. Dotted lines are sequence neighbor probabilities and solid lines are the fraction of bonds clustered.

# S5 System Parameters and Characteristic Timescale

Additional results obtained from variations to initial monomer density, non-bonded attraction strength, solvent viscosity, and activation energy are provided in Fig. S9 below. In order to map these results to the characteristic timescales as discussed in Fig. 3 in the main text, we first expressed these characteristic timescales in terms of the viscosity and activation energy parameters varied.

For the reactive timescale, $\tau_R$, we take the simplest definition[S11] of $\tau_R \equiv \frac{1}{k_{\mathrm{eff}}}$, where $k_{\mathrm{eff}}$ is the effective polymerization rate constant. In previous work,[S6] we have worked out an expression for $k_{\mathrm{eff}}$ for our system in terms of the relative activation energies, $E_{\mathrm{A}}^{ij}$, and the Arrhenius pre-exponential factors, $A_{ij}$, between each of the $i$,$j$ monomer species pairs, namely:

$$k_{\mathrm{eff}} = A_{\mathrm{AA,BB}}\exp(-\beta E_{\mathrm{a}}^{\mathrm{AA,BB}}) + A_{\mathrm{AB}}\exp(-\beta E_{\mathrm{a}}^{\mathrm{AB}}). \tag{S4}$$

To define a diffusive timescale, $\tau_D$, we make use of the diffusion coefficient, $D$, obtained via the Stokes-Einstein relation along with the viscosity implicit in the Langevin equation we employ (discussed in Section S1.3 above), taking

$$D = \frac{k_{\mathrm{B}}T}{3\pi\eta d}. \tag{S5}$$

Here $kT$ is the thermal energy, $\eta$ is the solvent viscosity, and $d$ is the diameter of a single monomer unit (namely $d = 1\sigma$ in our reduced LJ unit system). From the diffusion coefficient, we define the characteristic diffusive timescale, $\tau_D$, in terms of the time for a monomer to diffuse it's own diameter, that is:

$$\tau_D \equiv \frac{d^2}{D} = \frac{3\pi\eta d^3}{k_{\mathrm{B}}T}. \tag{S6}$$

In addition to the response of block length distribution, nematic ordering, and dispersity to the timescales defined above, Fig. S8 shows similar shifts in the extent and progression of oligomer and oligomer-oligomer bonding behaviors of systems in response to these timescales.

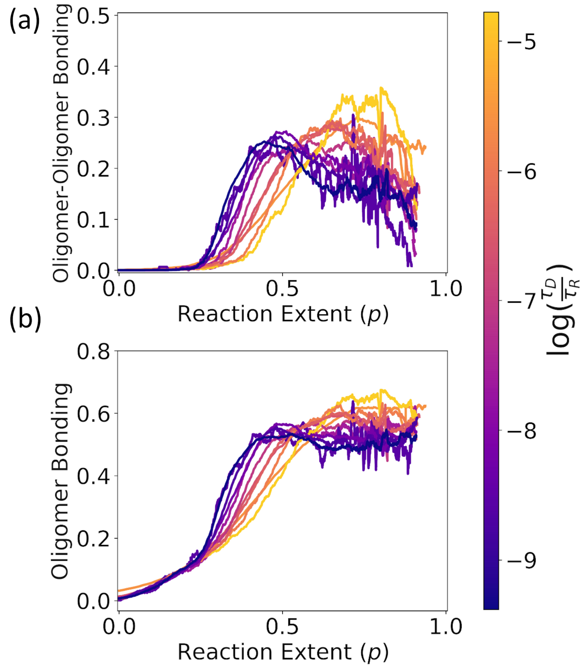


**Figure S8: Oligomer bonding and characteristic timescales.** Oligomer bond formation for the simulations explored in Fig. 3 in the main text. (a) Fraction of total new bond formation which is the result of oligomer bonding, i.e., of any chain of length $\geq 3$ forming a bond, as a function of reaction extent. (b) Fraction of total new bond formation which is the result of oligomer-oligomer bonding, namely both reacting chains are of length $\geq 3$ forming a bond, as a function of reaction extent. In both (a) and (b), coloration indicates the value of $\log(\tau_{\mathrm{D}}/\tau_{\mathrm{R}})$ the logarithm of the ratio of diffusive and reactive timescales for the simulation, with the dashed line indicating standard simulation conditions.

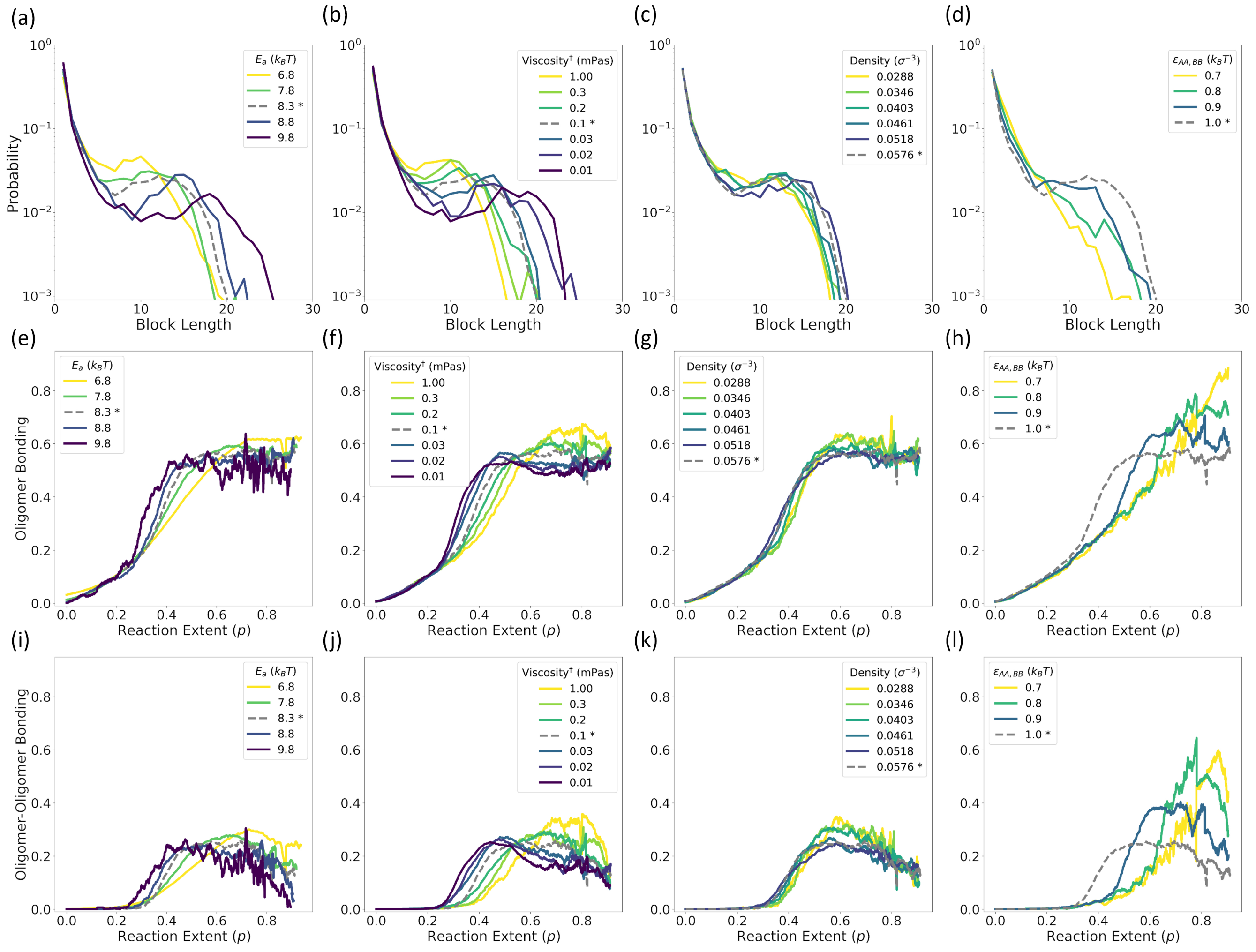


**Figure S9: Block length distributions and bonding populations vary with activation energy, viscosity, monomer density, and attraction strength.** (a-d) Block length distributions of all-**A** or all-**B** blocks are shown for the copolymerization of stiff chains ($l_p = 16.5$) at a reaction extent of $p = 0.9$. (e-h) Fraction of total new bond formation involving oligomers, as a function of reaction extent. Oligomers are defined here as chains of length three or longer. (i-l) Fraction of total new bond formation which is the result of oligomer-oligomer bonding, as a function of reaction extent. Results are shown here for variations in (a,e,i) activation energy, (b,f,j) solvent viscosity, (c,g,k) initial monomer densities, and (d,h,l) like-monomer attraction strengths. The gray, dashed lines in each plot, indicated with * in the legends, are identical and display the block length distribution or oligomer-oligomer bonding fraction found under the standard conditions described in Ref. S2. Bonding events are taken from three independent simulation trials per parameter set, and each distribution is obtained from three independent simulation trials at a reaction extent $p = 0.9$.

# S6 Characteristic Length in Homopolymer System

In order to verify that the characteristic length behavior we observed occurred irrespective of the phase-separation of comonomer species, we also ran homopolymer simulations under the same conditions as our standard copolymer simulation trials. The resulting chain length distributions, sampled from three independent simulation trials, are shown alongside the standard copolymer simulation in Fig. S10.

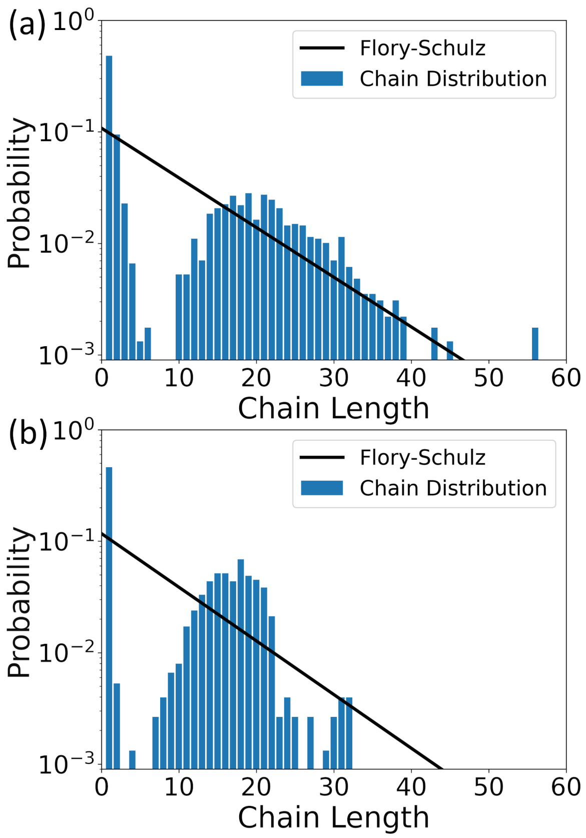


**Figure S10: Chain length distribution shift persists throughout polymer composition changes.** The observed chain length distribution at $p = 0.9$ for polymers with $l_p = 16.5$ where $\varepsilon_{\mathrm{AA,BB}} = k_{\mathrm{B}}T$ and $\varepsilon_{\mathrm{AB}} = 0$, for (a) copolymer with equal fraction of monomer species **A** and **B**, and (b) a homopolymer of species **A** (equivalent to a homopolymer of **B** by symmetry). In both (a) and (b) the expected Flory-Schulz distribution is also shown.

# References


(S1) Zhang, Z.; DuBay, K. H. Modeling the Influence of Emergent and Self-Limiting Phase Separations among Nascent Oligomers on Polymer Sequences Formed during Irreversible Step-Growth Copolymerizations. *Macromolecules* **2019**, *52*, 5480–5490.

(S2) Zhang, Z.; DuBay, K. H. The Sequence of a Step-Growth Copolymer Can Be Influenced by Its Own Persistence Length. *J. Phys. Chem. B* **2021**, *125*, 3426–3437.

(S3) Thompson, A. P.; Aktulga, H. M.; Berger, R.; Bolintineanu, D. S.; Brown, W. M.; Crozier, P. S.; in 't Veld, P. J.; Kohlmeyer, A.; Moore, S. G.; Nguyen, T. D.; Shan, R.; Stevens, M. J.; Tranchida, J.; Trott, C.; Plimpton, S. J. LAMMPS - A Flexible Simulation Tool for Particle-Based Materials Modeling at the Atomic, Meso, and Continuum Scales. *Comp. Phys. Comm.* **2022**, *271*, 108171.

(S4) Flory, P. J. *Principles of Polymer Chemistry*; Cornell University Press: Ithaca, New York, 1953; Chapter 3, pp 69–102.

(S5) Hamblin, R. L.; Nguyen, N. Q.; DuBay, K. H. Selective Solvent Conditions Influence Sequence Development and Supramolecular Assembly in Step-Growth Copolymerization. *Soft Matter* **2022**, *18*, 943–955.

(S6) Nguyen, N. Q.; Hamblin, R. L.; DuBay, K. H. Emergent Sequence Biasing in Step-Growth Copolymerization: Influence of Non-Bonded Interactions and Comonomer Reactivities. *J. Phys. Chem. B* **2022**, *126*, 6585–6597.

(S7) Dobrushin, R. L. Prescribing a System of Random Variables by Conditional Distributions. *Theory of Probab. & Its Appl.* **1970**, *15*, 458–486.

(S8) Vallender, S. S. Calculation of the Wasserstein Distance Between Probability Distributions on the Line. *Theory of Probab. & Its Appl.* **1974**, *18*, 784–786.

(S9) McInnes, L.; Healy, J.; Astels, S. HDBSCAN: Hierarchical Density Based Clustering. *JOSS* **2017**, *2*.

(S10) Campello, R. J. G. B.; Moulavi, D.; Sander, J. Density-Based Clustering Based on Hierarchical Density Estimates. Advances in Knowledge Discovery and Data Mining. Berlin, Heidelberg, 2013; pp 160–172.

(S11) Wartha, E.-M.; Bösenhofer, M.; Harasek, M. Characteristic Chemical Time Scales for Reactive Flow Modeling. *Combust. Sci. Technol.* **2021**, *193*, 2807–2832.
